# Volume electron microscopy in injured rat brain validates white matter microstructure metrics from diffusion MRI

**DOI:** 10.1162/imag_a_00212

**Published:** 2024-07-02

**Authors:** Ricardo Coronado-Leija, Ali Abdollahzadeh, Hong-Hsi Lee, Santiago Coelho, Benjamin Ades-Aron, Ying Liao, Raimo A. Salo, Jussi Tohka, Alejandra Sierra, Dmitry S. Novikov, Els Fieremans

**Affiliations:** Bernard and Irene Schwartz Center for Biomedical Imaging, Department of Radiology, New York University School of Medicine, New York, NY, United States; Athinoula A. Martinos Center for Biomedical Imaging, Department of Radiology, Massachusetts General Hospital, Harvard Medical School, Boston, MA, United States; A.I. Virtanen Institute for Molecular Sciences, University of Eastern Finland, Kuopio, Finland

**Keywords:** diffusion MRI, 3d electron microscopy, biophysical modeling, microstructure, white matter, traumatic brain injury

## Abstract

Biophysical modeling of diffusion MRI (dMRI) offers the exciting potential of bridging the gap between the macroscopic MRI resolution and microscopic cellular features, effectively turning the MRI scanner into a noninvasive *in vivo* microscope. In brain white matter, the Standard Model (SM) interprets the dMRI signal in terms of axon dispersion, intra- and extra-axonal water fractions, and diffusivities. However, for SM to be fully applicable and correctly interpreted, it needs to be carefully evaluated using histology. Here, we perform a comprehensive histological validation of the SM parameters, by characterizing white matter (WM) microstructure in sham and injured rat brains using volume electron microscopy and *ex vivo* dMRI. Sensitivity is evaluated by how well each SM metric correlates with its histological counterpart, and specificity by the lack of correlation with other, non-corresponding histological features. Compared to previously developed SM estimators with constraints, our results show that SMI is the most sensitive and specific. Furthermore, we derive the functional form of the fiber orientation distribution based on its exponentially decreasing rotational invariants. This comprehensive comparison with histology may facilitate the clinical adoption of *in vivo* dMRI-derived SM parameters as biomarkers for neurological disorders.

## 1 Introduction

Diffusion MRI (dMRI) has the remarkable ability (Alexander et al., 2017; Kiselev, 2017; Novikov et al., 2019) to probe the Brownian motion of water molecules on length scales comparable to cellular structures (∼10 μm), two orders of magnitude smaller than the standard clinical MRI resolution (∼1 mm). In this way, dMRI carries information about the cellular-level architecture restricting the diffusion of water molecules, and offers the exciting potential to characterize tissue microstructure *in vivo* and non-invasively in clinical settings (Jelescu & Budde, 2017). However, dMRI signals are indirect measures of tissue structural properties, and the task of identifying and extracting the relevant information remains an active field of research (Alexander et al., 2017; Jelescu & Budde, 2017; Novikov, Kiselev, et al., 2018; Novikov et al., 2019; Weiskopf et al., 2021).

Biophysical modeling of the dMRI signal strives to provide metrics that are not only sensitive but also specific to the underlying tissue microstructure (Jelescu & Budde, 2017; Novikov, Kiselev, et al., 2018). Biophysical models rely on assumptions (they “draw a picture”) about the geometry of the tissue that restricts the diffusion of the water. If these assumptions accurately capture the relevant properties that are encoded in the dMRI signal, such biophysical models can bridge the gap between the millimeter macroscopic MRI voxel resolution and the micrometer-level tissue architecture.

**Fig. 1.**
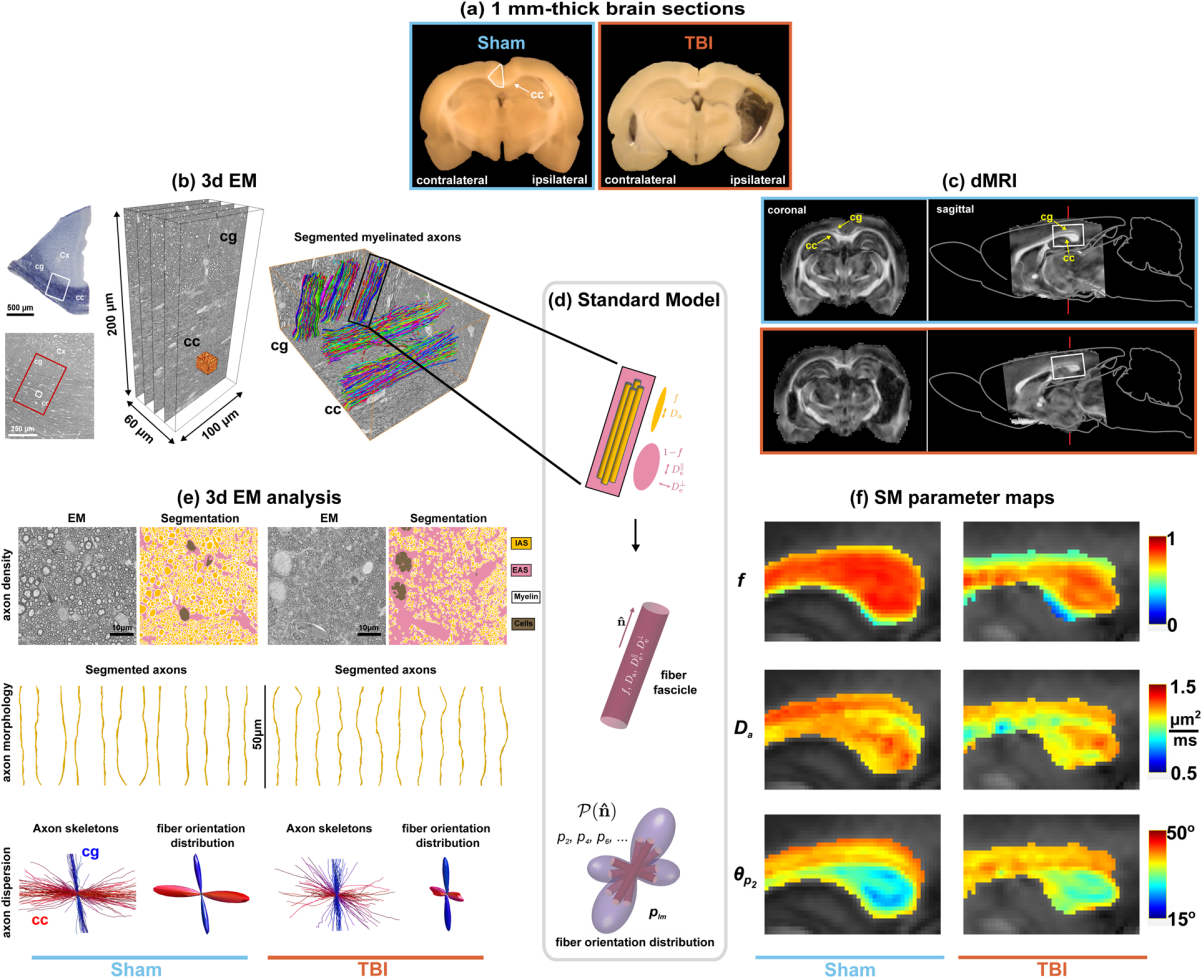
SM parameter maps reveal specific microstructure properties that correspond to 3d EM histology. (a) Brain sections (1 mm-thick) of sham-operated and TBI rats. (b) Location of the 3d EM samples within sham-operated rat brain and automatically segmented myelinated axons. (c) FA maps of rat brains in (a), indicating the corpus callosum (cc) and cingulum (cg) at the location where the 3d EM samples were collected, brain sagittal contours adapted from (Swanson, 2018). (d) In the Standard Model, a fiber fascicle is modeled by an intra-axonal compartment, in which axons are represented by cylinders with zero-radius, and an extra-axonal compartment represented by an axially symmetric tensor (see Section 2). These compartments are described by parameters f, $D_{\text{a}}$, $D_{\text{e}}^{∥}$ and $D_{\text{e}}^{⊥}$. One voxel contains multiple orientationally dispersed fascicles that contribute to the directional dMRI signal according to their FOD $P(\hat{n})$. (e) EM images transverse to the axons of the cingulum for the rat brains in (a), with segmented IAS, EAS, and myelin. Representative segmented 3d axons are also shown. Using skeletons from thousands of segmented axon, FOD for each axon population in the volumes were constructed. Reduction in axon density, changes in axon morphology, and increase in axon dispersion can be observed for the TBI animal with respect to the sham-operated animal. (f) dMRI-derived SM parametric maps reveal specific differences between sham and TBI that are observed in corresponding 3d EM histology.

In brain white matter (WM), at sufficiently long diffusion times, the dMRI signal of an elementary WM fiber segment, or *fascicle*, is modeled by two non-exchanging anisotropic Gaussian compartments (Novikov et al., 2019): an intra-axonal compartment in which axons are represented by impermeable cylinders with zero radius (Veraart et al., 2019); and an extra-axonal compartment represented by an axially symmetric tensor aligned with the fascicle. Given sufficient information in the dMRI measurements, a third, isotropic compartment could be considered. A macroscopic MRI voxel usually contains multiple dispersed fiber fascicles that contribute to the directional dMRI signal according to their orientation distribution. This general picture, illustrated in Figure 1d and mathematically defined in Section 2, is known as the *Standard Model* (Novikov et al., 2019) (SM) of diffusion in WM, as it unifies many previously proposed biophysical models relying on similar assumptions (Coelho et al., 2022; Fieremans et al., 2011; Jelescu et al., 2015a; Jespersen et al., 2007, 2018; Kaden et al., 2016; Kroenke et al., 2004; Lampinen et al., 2020; Novikov, Veraart, et al., 2018; Reisert et al., 2017; Veraart et al., 2018; Zhang et al., 2012).

In this work, we performed a comprehensive histological validation of the SM parameters, by characterizing brain WM microstructure on sham-operated and traumatic brain injury (TBI) rats, using volume (3d) electron microscopy (EM) and *ex vivo* dMRI. The 3d nature of our histological analysis, the variety of the EM samples (corpus callosum, cingulum, in the ipsi- and contralateral sides, for TBI and sham-operated animals), and the large number of segmented 3d axons (in the order of ten thousand per sample) all together enabled an unprecedented characterization of tissue microstructure. For the dMRI analysis, we estimated the SM parameters using four commonly used approaches: white matter tract integrity (WMTI) (Fieremans et al., 2011), neurite orientation dispersion and density imaging (NODDI) (Zhang et al., 2012), spherical mean technique (SMT) (Kaden et al., 2016), and standard model imaging (SMI) (Coelho et al., 2022; Novikov, Veraart, et al., 2018; Reisert et al., 2017). We refer to these as *SM estimators*, as they target the same biophysical SM parameters, yet differ in the additional constraints (see Table 1) and ways of performing model fitting (max-likelihood vs. machine learning). Our results show that while all these estimators provided SM parameters that correlate significantly (p<0.05) with their histological counterparts, indicating sensitivity, we found that SMI shows the highest specificity by presenting smallest cross-correlations with other, non-corresponding histological features. This comparison of SM parameters against 3d EM validates SM metrics as specific tissue biomarkers valuable for clinical applications and neuroscience research, as well as reveals the strengths and limitations of the chosen estimator.

**Table 1.** Standard model parameter estimators.

Method	Independent parameters	Parameter constraints	FOD
**WMTI** (Fieremans et al., 2011)	$f,D_{\text{a}},D_{\text{e}}^{∥},D_{\text{e}}^{⊥}$	$D_{\text{a}}\leq D_{\text{e}}^{∥}$	Coherently aligned fibers
**NODDI** (Zhang et al., 2012)	$f,f_{\text{w}},\kappa$	$D_{\text{a}}=D_{\text{e}}^{∥}=0.6\;\mu m^{2}/\text{ms}$ $D_{\text{e}}^{⊥}=\;D_{\text{e}}^{∥}\cdot \left(1-f\right)$	Watson distribution
**SMT** (Kaden et al., 2016)	$f,D^{∥}$	$D^{∥}=D_{\text{a}}=D_{\text{e}}^{∥}$ $D_{\text{e}}^{⊥}=D^{∥}\cdot \left(1-f\right)$	Factored out by spherical mean
**SMI** (Coelho et al., 2022; Reisert et al., 2017)	$f,D_{\text{a}},D_{\text{e}}^{∥},D_{\text{e}}^{⊥},f_{w},p_{lm}$	Unconstrained	Unconstrained

**Fig. 2.**
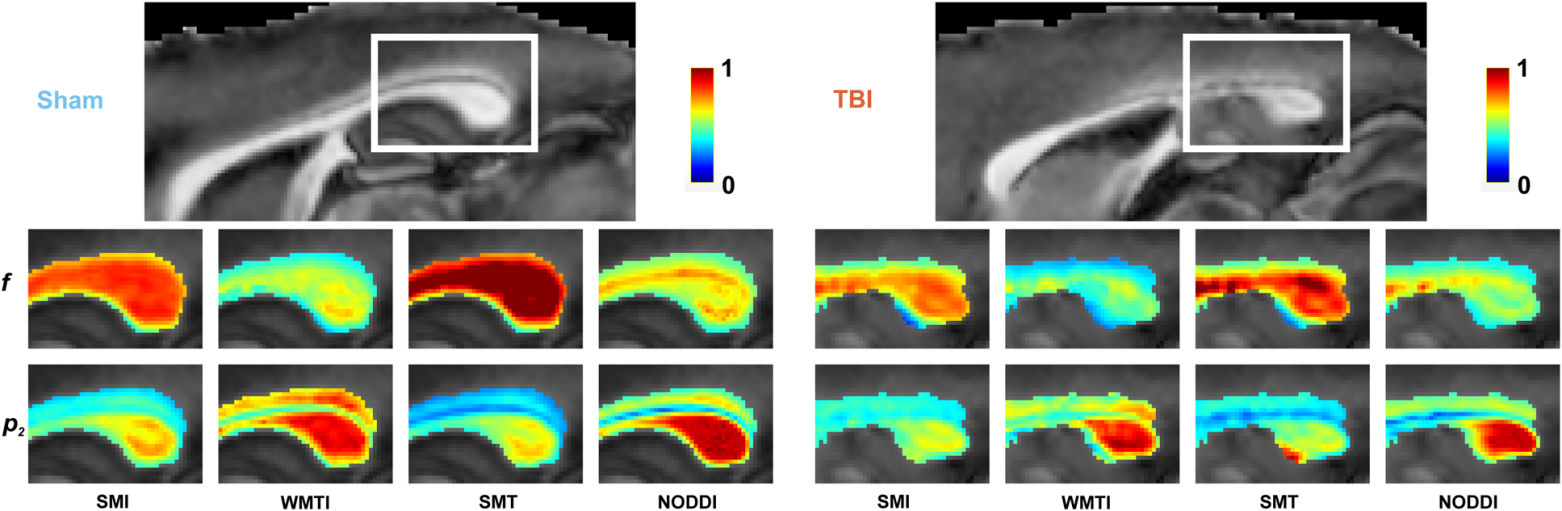
Standard Model parameter maps from different estimators. Maps for f (intra-axonal water fraction) and $p_{2}$ (inversely related to dispersion) from SMI, NODDI, WMTI, and SMT for a sham-operated and a TBI animal. Changes in TBI with respect to sham, such as the reduction in f and $p_{2}$, agree with histology for all methods. However, evident quantitative differences between their maps are observed.

**Fig. 3.**
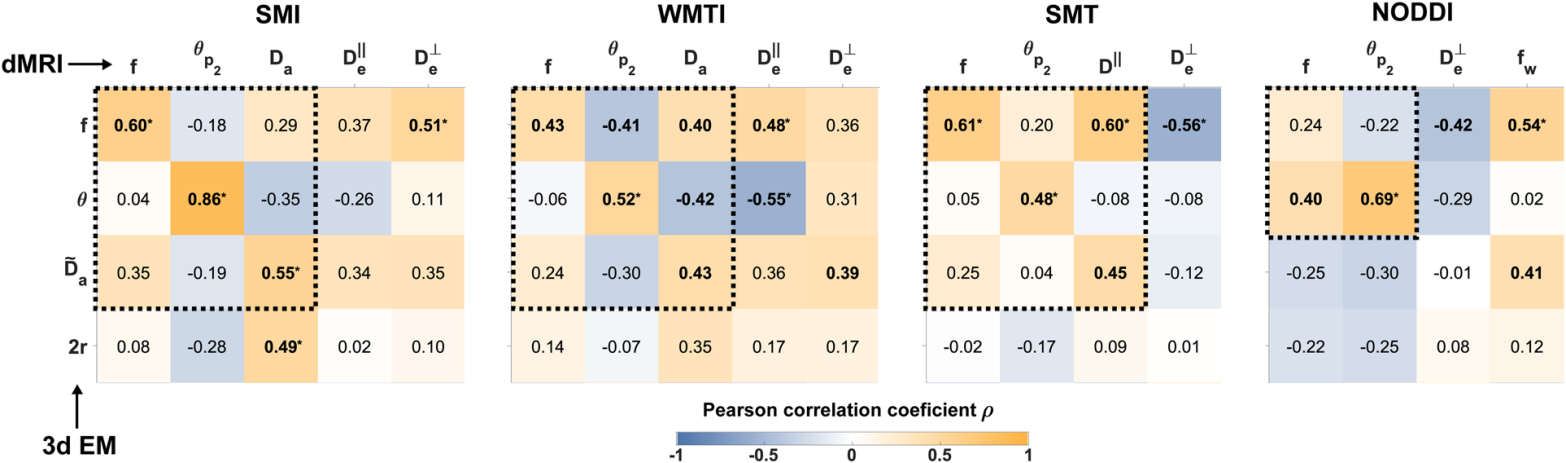
Comparison between dMRI and 3d EM derived metrics. For each estimator, matrices show the Pearson correlation coefficients comparing 3d EM histology-derived metrics (rows) against dMRI estimated SM parameters (columns), as plotted in Figure 4. Significant correlations (p<0.05) are highlighted in bold, where * indicates that significance remains after adjusting for multiple comparisons using the false discovery rate. Supplementary Figures S3–S6 provide the *p*-values and 95% confidence intervals. Submatrices for corresponding dMRI and 3d EM metrics, in which significant correlations are expected only in the diagonal, are highlighted by dashed lines. Similar values were obtained when using bootstrapping and permutation testing for the computation of the correlations and the *p*-values, respectively, as shown in Supplementary Figure S8.

**Fig. 4.**
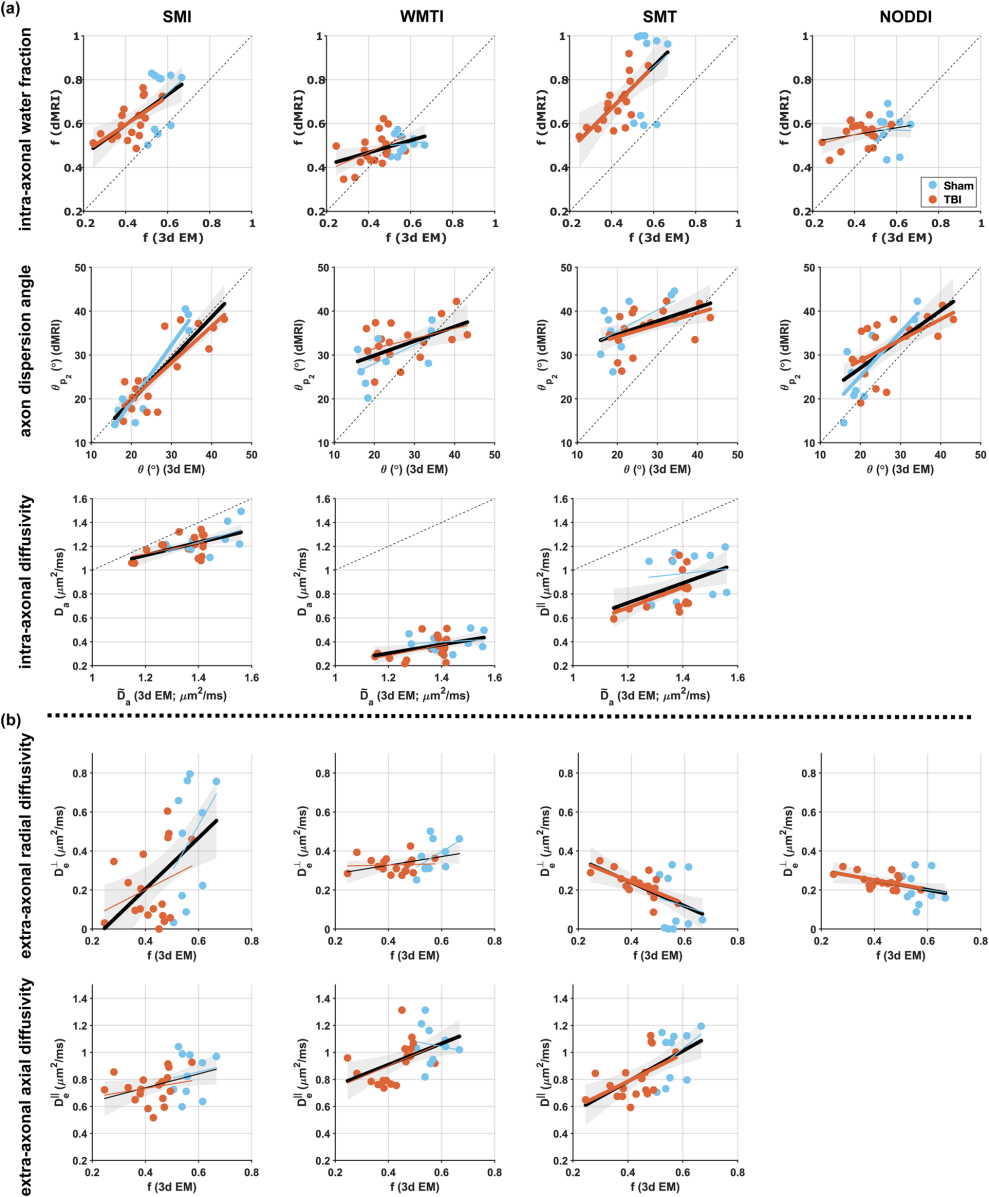
Scatter plots of dMRI-derived parameters against histology metrics. Linear regressions are shown using all (black lines and their 95% confidence intervals), TBI-only (red lines), and sham-only (blue lines) samples. Thicker lines indicate a correlation with p<0.05. The numerical values (using all samples) of the Pearson correlations coefficients are shown in Figure 3; their *p*-values and 95% confidence intervals are listed in Supplementary Figures S3–S6. (a) SM parameters f, $D_{\text{a}}$, and $\theta_{p_{2}}$ from SMI, WMTI, SMT, and NODDI compared against their corresponding 3d EM metrics. (b) SM extra-axonal diffusivities $D_{\text{e}}^{⊥}$ and $D_{\text{e}}^{∥}$ compared against histological f.

Although all these approaches aim to estimate the same SM parameters, the main differences between them are the number of independent parameters (second column), the constraints/interdependencies imposed on parameters (third column), the functional form of the FOD (fourth column), as well as different strategies for estimation (WMTI: linear diffusion and kurtosis tensors fit followed by explicit analytical formulas based on kurtosis tensor parameters; NODDI and SMT: max-likelihood; SMI: machine-learning approach based on the polynomial regression from signal’s rotational invariants to SM parameters). All these differences lead to different values in parametric maps, Figure 2, and correlations with histological counterparts, Figures 3 and 4.

The parameters provided by SM describe specific cellular features and could be a powerful tool for the study of pathological conditions. The *intra-axonal water fraction*f is a potential marker for axon density useful to quantify axonal loss (Coronado-Leija et al., 2021; Jelescu et al., 2016; Jespersen et al., 2010; Vestergaard-Poulsen et al., 2011), the *intra-axonal diffusivity*$D_{\text{a}}$ is a potential marker for axon morphology useful to quantify axonal injury such as beading (Budde & Frank, 2010; Hui et al., 2012; Lee et al., 2020), the *extra-axonal axial diffusivity*$D_{\text{e}}^{∥}$ is a potential marker for changes in the extra-axonal space such as inflammation, and the *extra-axonal radial diffusivity*$D_{\text{e}}^{⊥}$ is a potential marker for demyelination (Fieremans et al., 2012; Ginsburger et al., 2018; Jelescu et al., 2016).

Aside from the SM kernel parameters related to properties of fiber fascicles, the SM includes the *fiber orientation distribution*$P(\hat{n})$ (FOD) represented using spherical harmonics, which reflects the radial-angular connection (Novikov, Veraart, et al., 2018) in the diffusion q-space established by this basis. While the SM is agnostic to the FOD functional form, practically the maximal harmonic degree-l is determined by the diffusion weighting and the signal-to-noise ratio (Novikov, Veraart, et al., 2018). The properties of $P(\hat{n})$ can be quantified with FOD rotational invariants $p_{l}$, which are the energies of the FOD spherical harmonics coefficients $p_{lm}$ in each degree-l sector, see Eq. (3) in the Section 2. Here, the second-order rotational invariant $p_{2}$ is of special importance as it is inversely related (Novikov, Veraart, et al., 2018) to the average angle of dispersion of the axons θ via Eq. (6). $P(\hat{n})$ and $\theta_{p_{2}}$ are informative for structural brain connectivity, surgical planning, as well as to potentially assess pruning in neurodevelopment (Jelescu et al., 2015a).

The ability to accurately recover all SM parameters would reveal the wealth of WM tissue microstructure properties encoded in a relatively low-resolution *in vivo* dMRI data. Large initiatives are currently underway to collect extensive *in vivo* dMRI human brain data sets such as the Human Connectome Project (Glasser et al., 2016), the UK Biobank (Miller et al., 2016), the Alzheimer’s Disease Neuroimaging Initiative (Jack et al., 2008), and the Adolescent Brain Cognitive Development (Casey et al., 2018). Biophysical modeling combined with wide-scale *in vivo* dMRI measurements provides an unprecedented opportunity to study human brain changes in development (Jelescu et al., 2015a; Liao et al., 2024; Lynch et al., 2020; Mah et al., 2017), aging (Beck et al., 2021; Benitez et al., 2018; Cox et al., 2016), and disease (Benitez et al., 2014; de Kouchkovsky et al., 2016; Fieremans et al., 2013; Fu et al., 2019; Granberg et al., 2017; Hui et al., 2012; Johnson et al., 2021; Liao et al., 2024; Muller et al., 2021; Palacios et al., 2020; Wang et al., 2019).

## 2 Methods

All animal procedures were approved by the Animal Care and Use Committee of the Provincial Government of Southern Finland and performed according to the guidelines set by the European Community Council Directive 86/609/EEC.

### 2.1 Traumatic brain injury model

Five adult male Sprague-Dawley rats (10 weeks old, weights between 320 and 380 g, Harlan Netherlands B.V., Horst, Netherlands) were used in the study. The animals were housed in a room (22 ± 1°C, 50–60% humidity) with a 12 h light/dark cycle and free access to food and water.

TBI was induced by lateral fluid percussion injury in three rats (Kharatishvili et al., 2006). Rats were anesthetized with a single intraperitoneal injection (6 ml/kg) of a mixture of sodium pentobarbital (58 mg/kg), magnesium sulfate (127.2 mg/kg), propylene glycol (42.8%), and absolute ethanol (11.6%). Then, a craniotomy (5 mm diameter) was performed between bregma and lambda on the left convexity (anterior edge 2.0 mm posterior to bregma; lateral edge adjacent to the left lateral ridge). Lateral fluid percussion injury was induced by a transient fluid pulse impact (21–23 ms) against the exposed intact dura using a fluid-percussion device. The impact pressure was adjusted to 3.2–3.4 atm to induce a severe injury. The sham operation on the other two rats included all the surgical procedures except the impact. See Salo et al. (2018) and Abdollahzadeh et al. (2019) for detailed information about the animal model.

Five months after TBI or sham operation, the rats were transcardially perfused using 0.9% NaCl for 2 min followed by 4% paraformaldehyde (PFA). The brains were removed from the skull, post-fixed in 4% PFA/1% glutaraldehyde overnight at 4°C, and then placed in 0.9% NaCl for at least 12 h to remove excess PFA.

### 2.2 Ex vivo dMRI imaging

The extracted rat brains were scanned ex vivo at room temperature (21°C) in a vertical 9.4 T/89 mm magnet (Oxford Instruments PLC, Abingdon, UK) interfaced with a DirectDrive console (Varian Inc., Palo Alto, CA, USA) using a quadrature volume RF-coil (Ø = 20 mm; Rapid Biomedical GmbH, Rimpar, Germany) as both transmitter and receiver. During imaging, the brains were immersed in perfluoropolyether (Solexis Galden^®^, Solvay, Houston, TX, USA) to avoid signals from the surrounding area.

The dMRI data were acquired using a 3d spin-echo EPI sequence with four segments and resolution 0.150 × 0.150 × 0.150 mm^3^ (data matrix 128 × 96 × 96, FOV 19.2 × 14.4 × 14.4 mm^3^). TE/TR = 35/1000 ms. The acquisition comprised a total of 132 volumes that consisted of 3 sets of 43 monopolar directions uniformly distributed on the half-sphere with diffusion weighting (b-values) of 2, 3, and 4 ms/μm^2^, pulse length δ=6 ms, inter-pulse duration Δ=11.5 ms, and three non-diffusion-weighted images ($b_{0}$), interleaving one $b_{0}$ before each b-shell.

### 2.3 3d EM imaging

After the ex vivo dMRI acquisitions, brains were placed in 0.9% NaCl for at least 4 h to remove excess perfluoropolyether and then sectioned into 1 mm-thick coronal sections with a vibrating blade microtome. For each brain, sections at 3.80 mm from bregma in the caudal direction were selected and further dissected into smaller samples containing the areas of interest (Fig. 1a, b). Ten WM samples, two from each brain, were collected: the ipsi- and contralateral of the cingulum and corpus callosum. The samples were stained using an enhanced protocol with heavy metals, dehydrated, and embedded in Durcupan ACM resin. Before mounting the specimens, the excess resin in the hardened tissue blocks was trimmed. After selecting the area of interest for imaging within the samples, the blocks were further trimmed into a pyramidal shape with a 1 × 1 mm^2^ base and an approximately 600 × 600 μm^2^ top (face), which assured the stability of the block while being cut in the microscope. The tissue was exposed on all four sides, bottom, and top of the pyramid. Silver paint was used to electrically ground the exposed block edges to the aluminum pins, except for the block face or the edges of the embedded tissue. The entire surface of the specimen was then sputtered with a thin layer of platinum coating to improve conductivity and reduce charging during the sectioning process.

The blocks were imaged in a scanning electron microscope (Quanta 250 Field Emission Gun; FEI Co., Hillsboro, OR, USA) and equipped with the 3View system (Gatan Inc., Pleasanton, CA, USA) using a backscattered electron detector (Gatan Inc.). The face of the blocks was in the x-y plane, and the cutting was in z direction. All blocks were imaged using a beam voltage of 2.5 kV and a pressure of 0.15 Torr. After imaging, Microscopy Image Browser (MIB; http://mib.helsinki.fi) was used to process and align the EM image stacks.

Two datasets were acquired simultaneously at low- and high resolution consistently at one specific location in the white matter in both the ipsi- and contralateral hemispheres for all sham-operated and TBI animals as shown in Figure 1b. The low-resolution datasets were imaged from large tissue volumes of 200  ×  100  ×  60 μm^3^ with resolution 50 × 50 × 50 nm^3^. Two-thirds of the acquired volumes correspond to the corpus callosum and one-third to the cingulum. The corpus callosum region in the ipsilateral side for one rat was not collected as the quality of the staining of the tissue was not good enough for imaging. The high-resolution datasets were imaged from small tissue volumes of 15 × 15 × 15 μm^3^ on the corpus callosum with resolution 15 × 15 × 50 nm^3^. For detailed information about 3d EM tissue preparation, acquisition, and pre-processing, see Salo et al. (2018) and Abdollahzadeh et al. (2019).

### 2.4 3d EM segmentation

Myelin, myelinated axons, and cell nuclei were automatically segmented from the low-resolution datasets using the deep-learning-based pipeline DeepACSON (Abdollahzadeh, Belevich, et al., 2021; Abdollahzadeh, Sierra, et al., 2021) (https://github.com/aAbdz/DeepACSON), which is a method suited for the challenging task of segmenting large datasets with limited visualization of the cell membranes caused by low resolution.

First, the high-resolution datasets (small tissue volumes) were segmented using the ACSON pipeline (Abdollahzadeh et al., 2019), which is an automated method that integrates edge detection and seeded region growing algorithms in 3d, refining the segmentation with the SLIC (Simple Linear Iterative Clustering) supervoxel technique. These high-resolution images and their corresponding segmentations were down-sampled to match the low-resolution images (large tissue volumes) and used as human-annotation-free training sets for the DeepACSON pipeline (Abdollahzadeh, Belevich, et al., 2021). DeepACSON uses two convolutional neural networks to compute probability maps of myelin, myelinated axons, mitochondria, cell nuclei, and cell nuclei membranes. Then, these probability maps are binarized, and the segmentations are refined. Because of the low resolution of the images, the myelinated axons are prone to under-segmentation; for this reason, the segmented axons are rectified using a cylindrical shape decomposition method (Abdollahzadeh, Sierra, et al., 2021), and finally a support vector machine is used to exclude non-axonal segmented objects. For detailed information about the segmentation of these 3d EM samples, see Abdollahzadeh, Belevich, et al. (2021) and Abdollahzadeh, Sierra, et al. (2021).

Further proofreading was performed by considering only myelin attached to the axons, thus removing spurious segmented objects in the extra-axonal space that were labeled as myelin. For the computation of volume fractions, all segmented axons were considered, but for the quantification of axon diameter, cross-sectional area variation, and the fiber orientation distribution, only axons with lengths larger than 10 μm were used (accounting for more than 90% of the intra-axonal space).

### 2.5 Microstructural metrics derived from 3d EM

Axon population properties were obtained following the approach in Lee et al. (2019) (https://github.com/NYU-DiffusionMRI/RaW-seg). To facilitate the computation of axon cross-sectional areas, each axon main direction was first aligned to the z-axis. Then, the axon skeleton was created as the line connecting the center of mass of each slice. In order to avoid biases due to oblique cross-sections at the extremes caused by the inclination of the axons, 1 μm was removed at the two ends of each axon.

#### 2.5.1 Fiber orientation distribution

Fiber orientation distributions for each axon population were computed using the corresponding skeletons of the aligned axons by rotating them back to the original axon main directions and projecting their tangent vectors on a 3d triangulated spherical surface (Lee et al., 2019). In this way, a histogram $P(\hat{n})$ was created, normalized (Novikov, Veraart, et al., 2018) onto $\int \text{d}\hat{n}P(\hat{n})\equiv 1$, where $\text{d}\hat{n}\equiv {\text{dΩ}}_{\hat{n}}/4\pi$ and ${\text{dΩ}}_{\hat{n}}\equiv \text{sin }\theta \text{d}\theta \text{d}\phi$ is the canonical area element on a unit sphere, such that $\int \text{d}\hat{n}\equiv 1$. To simulate coarse-grained effects caused by diffusion in the calculation of the FOD, the axon skeletons were smoothed by a Gaussian kernel with variance $\sigma^{2}=L^{2}/4$ (Lee et al., 2019; Novikov et al., 2019), where $L=\sqrt{2Dt}$ is the diffusion length, $D=2\;\mu {\text{m}}^{2}/\text{ms}$, and t is the diffusion time (inter-pulse duration Δ) of the dMRI acquisition.

The average angle of deviation from the main axis or dispersion angle for each axon population was computed using (Lee et al., 2019):

$$\theta ={\text{cos}}^{-1}\sqrt{\langle {\text{cos}}^{2}\theta_{i}\rangle },$$ (1)

where the individual axon segment’s dispersion angle $\theta_{i}$ is the angle between the axon segment’s direction with respect to the main direction of the axon population.

Each histological FOD $P(\hat{n})$ was decomposed in the spherical harmonics (SH) basis:

$$P\left(\hat{n}\right)\approx 1+\sum_{l=2,4,...\;}^{l_{\text{max}}}\sum_{m=-l}^{l}p_{lm}Y_{lm}\left(\hat{n}\right),$$ (2)

where the SH coefficients are $p_{lm}\equiv 4\pi \int \text{d}\hat{n}Y_{lm}(\hat{n})P(\hat{n})$ and $Y_{lm}(\hat{n})$ are the standard SH normalized to $4\pi \int \text{d}\hat{n}$$|Y_{lm}(\hat{n}){|}^{2}\equiv 1$. Because of FOD antipodal symmetry, only even harmonic orders l are considered up to a maximum order of $l_{\text{max}}=16$.

Based on the SH decomposition of $P(\hat{n})$ in Eq. (2), FOD rotational invariants $p_{l}$ are defined as (Novikov, Veraart, et al., 2018; Reisert et al., 2017)

$$p_{l}^{2}=\frac{1}{N_{l}^{2}}\sum_{m=-l}^{l}|p_{lm}{|}^{2},\;\;0\leq p_{l}\leq 1,$$ (3)

where the normalization factor (Novikov, Veraart, et al., 2018)

$$N_{l}=\sqrt{4\pi \left(2l+1\right)}$$ (4)

has been chosen such that $0\leq p_{l}\leq 1$. The rationale for this normalization can be m=0 harmonics

$$Y_{l0}\left(\theta ,\phi \right)=\left(N_{l}/4\pi \right)P_{l}\left(\text{cos}\theta \right)$$ (5)

are proportional to the Legendre polynomials $P_{l}\left(x\right)\leq 1$; for an axially symmetric FOD pointing along z-axis, $p_{lm}=0$ for m≠0, and the only contribution to $p_{l}$ comes from $p_{l0}=4\pi \langle Y_{l0}(\hat{n})\rangle =N_{l}\langle P_{l}\left(\text{cos}\theta \right)\rangle$, yielding (Novikov, Veraart, et al., 2018) $p_{l}=\left|\langle P_{l}(\text{cos}\theta )\rangle \right|\leq 1$, where $\langle \ldots \rangle =\int \text{d}\hat{n}$$P(\hat{n})\ldots$ is the average over FOD. The value $p_{l}=1$ is attained for a fully aligned fiber bundle $P=2\delta \left({\left(\hat{n}\cdot \hat{z}\right)}^{2}-1\right)$, since $P_{l}\left(1\right)=1$.

Similar to Eq. (1), as a measure of the FOD dispersion we use $\langle {\text{cos}}^{2}\theta_{i}\rangle \to \langle {\text{cos}}^{2}\theta \rangle$, the variance of cosθ in the fiber basis over the FOD. In the fiber basis, assuming axial symmetry, $p_{l}=\left|p_{l0}\right|/N_{l}$, which allows us to define (Lee et al., 2019; Novikov, Veraart, et al., 2018) the dispersion angle based on the l=2 FOD invariant:

$$\theta_{p_{2}}\equiv {\text{cos}}^{-1}\sqrt{\frac{2p_{2}+1}{3}},$$ (6)

given that $P_{2}\left(x\right)=(3x^{2}-1)/2$. While a more precise dispersion angle would involve Eq. (6) with $p_{2}\to p_{20}/N_{2}$ in the fiber basis, the advantage of the definition in Eq. (6) is that $p_{2}$ can be calculated in any basis.

In this work, we validated the hypothesis that the FOD rotational invariants in Eq. (3) decrease exponentially with l:

$$p_{l}=\{\begin{array}{c} C\lambda^{l},\;\;l=2,\;4,\;\ldots ,\;\;0\leq \lambda \leq 1;\\ 1,\;\;l=0\;\left(normalization\right). \end{array}$$ (7)

While Eq. (7) was previously tested (Lee et al., 2019) using 320 segmented 3d axons from the corpus callosum of a normal mouse, we validated this scaling using a much larger number of segmented axons, in the order of ten thousand per sample, from the corpus callosum and cingulum of rats with and without TBI pathology. We also derived, in the Appendix, Eq. (A5) for the axially-symmetric FOD corresponding to Eq. (7), and compare it with the earlier proposed Watson (Zhang et al., 2012) and Poisson kernel (Reisert et al., 2017) FOD functional forms using FODs obtained from 3d EM.

#### 2.5.2 Intra-axonal volume fraction

Using the segmented 3d EM samples, the intra-axonal, extra-axonal, and myelin volumes were computed as the number of voxels covering each compartment. The dMRI-related intra-axonal volume fraction metric f was computed as the intra-axonal volume divided by the total volume of the sample, excluding the myelin compartment due to its short $T_{2}$ compared with the TE of the dMRI acquisition (MacKay et al., 1994), which makes it dMRI invisible.

#### 2.5.3 Intra-axonal diffusivity

The cross-sectional area A(z) and the diameter 2r≡$2\sqrt{A\left(z\right)/\pi }$ of a circle with the same area were computed for each slice perpendicular to the skeleton of the aligned axons. It has been shown in Lee et al. (2020) that the intra-axonal diffusivity $D_{\text{a}}$ is mainly affected by the along-axis variation of A(z). Recently, the exact axial tortuosity

$$\Lambda_{∥}=\frac{D_{0}}{D_{\text{a}}{|}_{t\to \infty }}=\langle \frac{\bar{A}}{A\left(z\right)}\rangle ,$$ (8)

was found (Abdollahzadeh et al., 2023) based on the Fick-Jacobs equation, where $\bar{A}=\langle A\left(z\right)\rangle$ is the mean cross-sectional area of the axon. This yields the prediction for $D_{\text{a}}$ in the long-time, t→∞ limit based on the unrestricted diffusivity $D_{0}$ in the axoplasm. For a perfect cylinder axon with no variations in A(z), $D_{\text{a}}=D_{0}$ for all times.

The undulation of the axons, determined by the ratio between their geodesic and euclidean lengths, or sinuosity, can also affect $D_{\text{a}}$. To account for this, we computed the dispersion angle $\theta_{\text{u}}$ caused only by undulations (Lee et al., 2020) using Eq. (1) on the axons aligned to the z-axis.

Assuming free diffusivity in the axoplasm for perfect cylinder axons ($D_{0}=D_{\text{w}}=2.0\;\mu {\text{m}}^{2}/\text{ms}$ for dMRI acquired at room temperature), the value of SM intra-axonal diffusivity $D_{a}$ in the EM sample can be predicted using Eq. (8), and $\theta_{\text{u}}$ computed with Eq. (1) on each aligned axon, aggregating the contribution for all of them:

$${\tilde{D}}_{\text{a}}=\sum_{k}w_{k}\frac{D_{\text{w}}}{\Lambda_{k}^{∥}}\cdot {\langle {\text{cos}}^{2}\theta_{\text{u}}\rangle }_{k},$$ (9)

where the weights $w_{k}$ are proportional to the axon volumes, with $\sum_{k}w_{k}\equiv 1$.

### 2.6 Standard Model

The Standard Model (Novikov et al., 2019) of the dMRI signal, for brain WM, in the direction $\hat{u}$ (Fig. 1d), can be written as:

$$S\left(b,\xi ,\hat{u}\right)=S_{0}\int_{\left|\hat{n}\right|=1}\text{d}\hat{n}\;P\left(\hat{n}\right)\;K\left(b,\xi ,\hat{u}\cdot \hat{n}\right).$$ (10)

This equation describes the dMRI signal $S\left(b,\xi ,\hat{u}\right)$ as a spherical convolution (Tournier et al., 2007) between the FOD $P(\hat{n})$ and the SM kernel $K\left(b,\xi ,\hat{u}\cdot \hat{n}\right)$, which represents an elementary fiber segment, and is the sum of at least two non-exchanging, intra- and extra-axonal Gaussian compartments:

$$K\left(b,\xi ,\hat{u}\cdot \hat{n}\right)=f\;\;e^{-bD_{\text{a}}{\left(\hat{u}\cdot \hat{n}\right)}^{2}}+\left(1-f\right)e^{-bD_{\text{e}}^{⊥}-b\left(D_{\text{e}}^{∥}-D_{\text{e}}^{⊥}\right){\left(\hat{u}\cdot \hat{n}\right)}^{2}},$$

where b is the diffusion weighting, $\hat{u}\cdot \hat{n}$ is the cosine of the angle between the symmetry axis of the SM kernel $\hat{n}$ and the measured direction $\hat{u}$, and ξ are the parameters of the SM kernel:

$$\xi =\left\{f,D_{\text{a}},D_{\text{e}}^{∥},D_{\text{e}}^{⊥}\right\}.$$ (11)

Given sufficient information in dMRI measurements, the fraction $f_{\text{w}}$ corresponding to the free water compartment $e^{-bD_{\text{w}}}$ could also be considered.

In the SH basis, the convolution between the FOD and the SM kernel in Eq. (10) becomes a product

$$s_{lm}\left(b,\xi \right)=p_{lm}\;K_{l}\left(b,\xi \right),$$ (12)

where

$$K_{l}\left(b,\xi \right)=\int_{0}^{1}\text{d}x\;K\left(b,\xi ,x\right)\;P_{l}\left(x\right)$$ (13)

are the kernel’s projections onto the Legendre polynomials $P_{l}\left(x\right)$ with even l. By constructing the rotational invariants of the dMRI signal, similar to Eq. (3),

$$s_{l}^{2}={\frac{1}{N}}_{l}\sum_{m=-l}^{l}|s_{lm}{|}^{2},$$ (14)

the FOD can be factored out (Novikov, Veraart, et al., 2018; Reisert et al., 2017), reducing the SM to a set of equations

$$s_{l}\left(b,\xi \right)=p_{l}\;K_{l}\left(b,\xi \right),$$ (15)

one per SH degree l.

The estimation of the SM parameters from conventional Pulsed-Gradient Spin-Echo (PGSE) dMRI is challenging because the problem is ill-posed (Jelescu et al., 2015b; Novikov, Veraart, et al., 2018). It has been shown that multi-dimensional dMRI acquisitions are needed for an accurate, precise, and robust estimation of all the SM metrics (Coelho et al., 2022; Lampinen et al., 2020; Novikov, Veraart, et al., 2018). Nonetheless, there is still a need to correctly estimate the SM parameters on the typical PGSE data used in this study, as the results can be extrapolated to the majority of clinical human studies that use similar dMRI protocols including the large multicenter dMRI datasets currently being collected (Casey et al., 2018; Glasser et al., 2016; Jack et al., 2008; Miller et al., 2016). Several strategies have been proposed to solve the degeneracy of the SM parameter estimation in PGSE data. The principal characteristics of these strategies, regarding estimated parameters and constraints, are summarized in Table 1.

*White matter tract integrity* (Fieremans et al., 2011) uses the diffusion and kurtosis tensors to derive formulas for the SM parameters; however, it is mostly valid for aligned axon populations. *Neurite orientation dispersion and density imaging* (NODDI) (Zhang et al., 2012) is the most widely used estimator for f and the FOD (as Watson distribution); however, it fixes the axial diffusivities, and constrains the extra-axonal radial diffusivity using the first-order tortuosity approximation (Szafer et al., 1995). *Spherical mean technique* (SMT) (Kaden et al., 2016) uses the spherical mean ($0^{th}$ order rotational invariant) on each shell of the dMRI signal to factor out the FOD and focus only in the estimation of the SM kernel parameters; however, it uses two of NODDIs strong assumptions. *Standard model imaging* (SMI) (Coelho et al., 2022; Reisert et al., 2017) uses a machine-learning approach to estimate kernel parameters from the system (15). The rotational invariants of the dMRI signal, Eq. (14) are mapped to the SM kernel parameters (11) via cubic polynomial regression. Common to machine-learning estimators, when the information in the data is insufficient, the estimation of the parameters can be biased by the training data.

Once the SM kernel parameters ξ are obtained, the FOD SH coefficients $p_{lm}$ can be estimated from Eq. (12). Also, the FOD SH rotational invariants $p_{l}$ can be directly computed from Eq. (15). In our study, SMI provided $p_{lm}$ directly. For NODDI, which assumes the FOD as a Watson distribution, in Eq. (A14), $p_{2}$ was obtained using its relation (Jespersen et al., 2018) to the concentration parameter κ, in Eq. (A15). Eq. (15) was used to compute $p_{2}$ for SMT. For WMTI, $p_{2}$ was directly computed from the intra-axonal diffusivity $D_{\text{a}}$ and the intra-axonal diffusion tensor eigenvalues $\lambda_{a1},\lambda_{a2},$ and $\lambda_{a3}$ estimated by WMTI, using the approach described in the appendix C of Novikov, Veraart, et al. (2018):

$$p_{2}^{2}=\frac{3}{2}\frac{1}{D_{\text{a}}^{2}}\sum_{i=1,2,3}{\left(\lambda_{ai}-\frac{D_{\text{a}}}{3}\right)}^{2}.$$

### 2.7 dMRI processing

In order to improve the precision and accuracy of the estimated SM maps, dMRI data were processed following the steps in the DESIGNER pipeline (https://github.com/NYU-DiffusionMRI/DESIGNER-v1) (Ades-Aron et al., 2018) adapted for *ex vivo* dMRI rat brains. Magnitude and phase volumes were reconstructed from the data to perform MP-PCA complex-denoising (Lemberskiy et al., 2020; Veraart et al., 2016), which has been shown to highly reduce the Rician noise floor. Images were also corrected for Gibbs-ringing (Kellner et al., 2015), and registration (Avants et al., 2008) was used to correct for geometric distortions induced by eddy currents, as well as image drift over the long acquisition period. WMTI maps were computed using tools provided by the DESIGNER pipeline (https://github.com/NYU-DiffusionMRI/DESIGNER-v1/tree/master/utils/). NODDI, SMT, and SMI maps were estimated with their respective toolboxes: SMT (https://github.com/ekaden/smt), NODDI (http://mig.cs.ucl.ac.uk/index.php?n=Tutorial.NODDImatlab), and SMI (https://github.com/NYU-DiffusionMRI/SMI).

For NODDI, the axial diffusivity was fixed to the suggested value for *ex vivo*, $D^{∥}=D_{\text{a}}=D_{\text{e}}^{∥}=0.6\;\mu {\text{m}}^{2}/\text{ms}$. For SMT and SMI, the upper bound for the diffusivities was set to the free diffusion of water at room temperature $∼2\;\mu {\text{m}}^{2}/\text{ms}$. For SMI, rotational invariants up to l=6 were used for parameter mapping. SM *free water fraction*$f_{\text{w}}$ metric can be estimated by SMI and NODDI; however, imaging was performed using b=2, 3, 4 ms/$\mu {\text{m}}^{2}$ at room temperature ($21^{{}^{\circ}}$C), for which free water signal is negligible (≈1.5% of $S_{0}$ for the lower b-vale). For this reason, for SMI we set $f_{\text{w}}=0$. However, for NODDI, $f_{\text{w}}$ was estimated as it was shown to improve the correlations of its other parameters. Manually delineated ROIs were drawn by identifying, in the diffusion tensor ellipsoids, the corpus callosum, cingulum, and their crossing region in the ipsi- and contralateral side at the location where the 3d EM samples were collected (Supplementary Fig. S1).

**Fig. 5.**
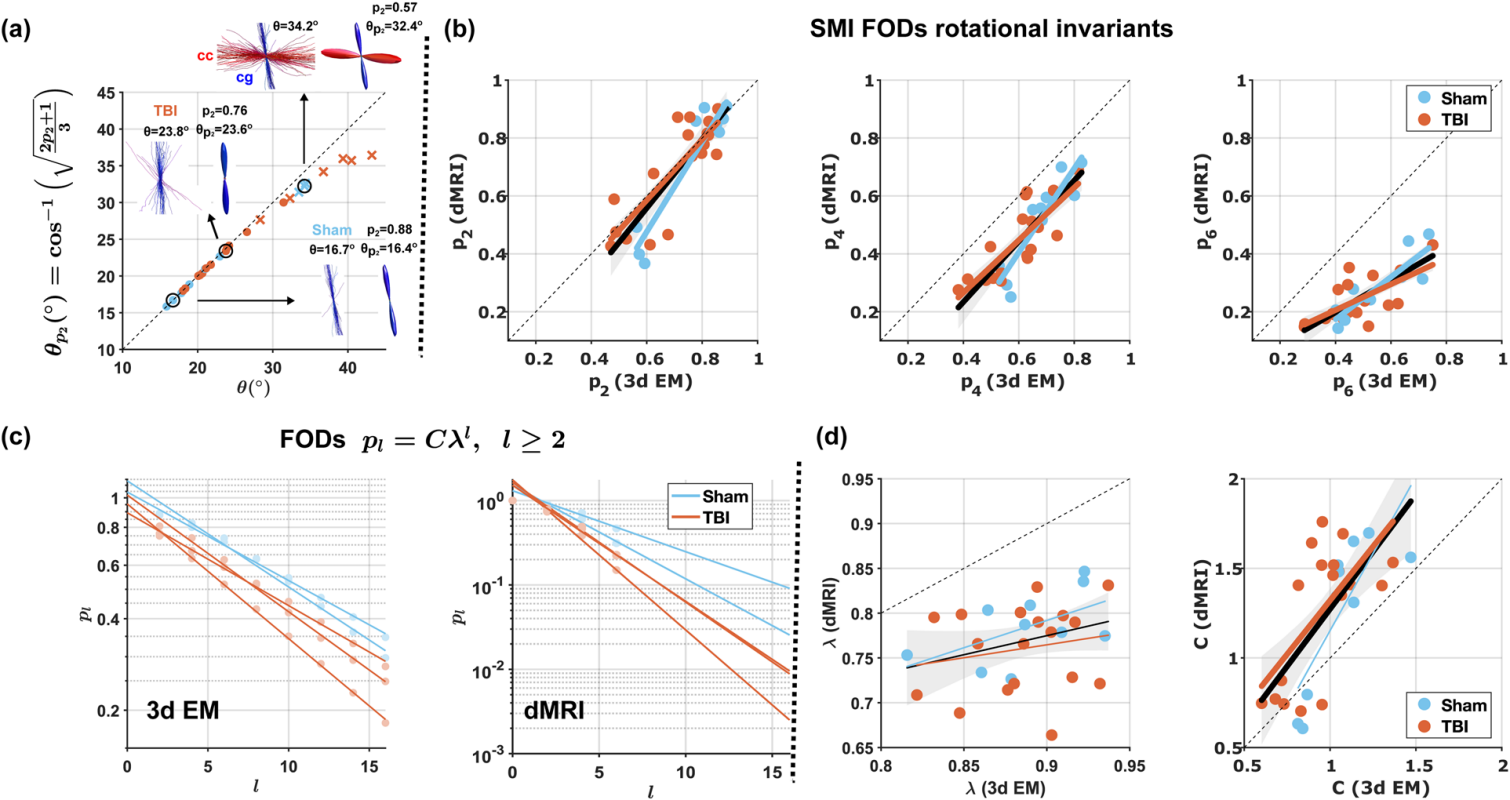
Dispersion angle θ, FOD rotational invariants $p_{l}$, and their exponential decay. (a) For 3d EM histology, dispersion angle $\theta_{p_{2}}$ (computed from $p_{2}$) corresponds to the true average angle of deviation θ of the axons with respect to the main direction of the axon population. The agreement is better for single bundle FOD (⋅ markers) than for crossing fibers (x markers). (b) FOD rotational invariants $p_{2}$, $p_{4}$, $p_{6}$ correlate with their histological counterparts. (c) FOD $p_{l}$ follow an exponential decay $p_{l}=C\lambda^{l}$ for l=2, 4,…. Examples from dMRI and 3d EM in the ipsilateral cingulum for all animals. (d) Comparison between dMRI (SMI) and 3d EM derived parameters C and λ.

FOD second-order rotational invariant $p_{2}$ is sensitive to axon dispersion, as shown in Figure 5a, and it is affected by complex fiber configuration such as crossing fibers in the voxel. In the dMRI volumes, we observed partial volume in the cingulum ROI which contained a small fraction of corpus callosum axons in the perpendicular direction. For this reason, the SM maps in Figures 1f and 2 show increased dispersion (higher $\theta_{p_{2}}$ and lower $p_{2}$) in the cingulum voxels with respect to the corpus callosum voxels, contrary to what it is observed in the 3d EM volumes. SMI estimates the full non-parametric FOD, from which we segmented the lobes corresponding to the different axon populations (Riffert et al., 2014), then from the segmented lobes we computed per-bundle rotational invariants $p_{l}$.

### 2.8 SM parameter evaluation using 3d EM metrics

Sensitivity and specificity were evaluated by comparing SM parameters f, $\theta_{p_{2}}$ and $D_{\text{a}}$ with their 3d EM counterparts on six ROIs: cc, cg, cc+cg for the ipsi- and contralateral hemispheres for each animal, as shown in Supplementary Figure S1. While histological f and θ were computed directly from the segmented axons, ${\tilde{D}}_{a}$ was predicted from histology, Eq. (9), by considering the axons axial tortuosity (Abdollahzadeh et al., 2023) $\Lambda_{∥}$ and the dispersion angle caused only by axon undulations (Lee et al., 2020) $\theta_{\text{u}}$, assuming free diffusivity $D_{\text{w}}=2\;\mu {\text{m}}^{2}/\text{ms}$ (at room temperature) for perfect cylinder axons. Influence of axon diameter 2r in the SM parameters was also evaluated.

### 2.9 Statistics

Pearson correlations (ρ), *p*-values (p), and 95% confidence intervals (CI) between each dMRI and 3d EM derived metric pairs were computed using MATLABs command **corrcoef**, which uses a t-test with N−2 degrees of freedom for *p*-values (N is the number of samples), and for the confidence intervals uses an asymptotic Gaussian distribution of $\frac{1}{2}\text{ln}\left(\frac{1+\rho }{1-\rho }\right)$, with an approximate variance equal to 1/​(N−3). Here, the total number of samples in the analysis is 28 instead of 30, as the corpus callosum part of the EM is missing, hence no cc and no cc+cg for one sham-operated animal. We corrected for multiple comparisons using the False Discovery Rate (FDR) computed with MATLABs command **mafdr** that uses the Benjamini–Hochberg procedure. Concordance correlation coefficients $\rho_{c}$ were also computed between SM dispersion angle $\theta_{p_{2}}$ and its histological counterpart θ, as agreement in their values is expected for these metrics in particular. It is important to mention that a significant correlation (p<0.05) does not necessarily imply causality, as a pathological condition such as TBI is very complex, with many microstructural changes happening simultaneously, and it is challenging to get the full picture of how a particular SM parameter is affected by them.

## 3 Results

The TBI rat brains showed visible damage due to the impact on 1 mm-thick brain sections (Fig. 1a) and corresponding fractional anisotropy (FA) maps (Fig. 1c), as opposed to sham-operated rat brains showing no visible damage. To reveal the corresponding changes caused by TBI at the microstructural scale, we automatically segmented the myelin and myelinated axons on 3d EM, as illustrated in Figure 1b. Figure 1e and Supplementary Figure S2 shows the reduction of axonal density, changes in axon morphology (diameter 2r, axial tortuosity $\Lambda_{∥}$ and undulations, as defined in Section 2), as well as an increase in axonal dispersion in the TBI sample. Remarkably, these microstructural changes were also detected non-invasively by ex vivo MRI, as shown on the SM maps in Figure 1f for the axon water fraction f, intra-axonal diffusivity $D_{\text{a}}$, and axon dispersion angle $\theta_{p_{2}}$.

Parametric maps obtained from the same ex vivo dMRI measurement but using different estimators (SMI, WMTI, NODDI, SMT) are compared in Figure 2 for f and $p_{2}$; its relation to dispersion angle via Eq. (6) is demonstrated in Figure 5a. Consistent changes are observed between sham-operated and TBI animals that are in qualitative agreement with the histology shown in Figure 1e and Supplementary Figure S2. Yet, the maps also demonstrate quantitative differences among the distinct estimators.

The diagonals of the dashed submatrices in Figure 3 indicate that all SM estimators provided metrics that correlated significantly (p<0.05) with their corresponding tissue properties derived from 3d EM histology, except for f from NODDI. This suggests good overall sensitivity of all SM estimators. Here, SMI achieved the highest correlations for the three SM metrics, with only SMT providing a similar correlation for f.

Regarding specificity, the off-diagonal elements of the dashed submatrices in Figure 3 show that only SMI had no significant correlations with non-corresponding histological features, while the other estimators revealed multiple spurious cross-correlations. Looking at each SM parameter individually (columns), dMRI f was specific to its histological counterpart for SMI, WMTI, and SMT, while for NODDI, f correlated with 3d EM θ. dMRI $\theta_{p_{2}}$ was specific to histological θ for SMI, SMT, and NODDI, while WMTI $\theta_{p_{2}}$ correlated also with 3d EM f. dMRI $D_{\text{a}}$ was specific to its predicted value from histology ${\tilde{D}}_{a}$ only for SMI, while for SMT, $D_{\text{a}}$ correlated also with 3d EM f, and for WMTI, $D_{\text{a}}$ correlated also with 3d EM f and θ. The overall highest specificity of SMI parameters can be understood by the lack of hard constraints and assumptions as opposed to the other estimators (see Table 1).

In the scatter plots of dMRI f against EM f of Figure 4a, agreement with the identity line was not expected. For one part, the resolution of the EM samples did not allow the segmentation of unmyelinated axons. Furthermore, dMRI data were acquired using a single TE, so SM f is $T_{2}$-weighted, while EM f is not. For these reasons, and assuming intra-axonal $T_{2}$ longer than extra-axonal $T_{2}$, (Tax et al., 2021; Veraart et al., 2018) an overestimation of dMRI f was expected.

On the other hand, agreement with the identity line was expected when comparing dMRI $\theta_{p_{2}}$ against EM θ in the scatter plots of Figure 4 (Georgiadis et al., 2018), assuming unmyelinated axons have similar dispersion to myelinated axons, and the fiber orientation distribution is unaffected by relaxation. Figure 4a shows better agreement with the identity line for $\theta_{p_{2}}$ from SMI than from the other methods, also indicated by its highest Pearson correlation (ρ) values shown in Figure 3 and its highest concordance correlation coefficients $\rho_{c}$, with respect to the other methods: SMI $\rho_{c}=0.850$, WMTI $\rho_{c}=0.332$, SMT $\rho_{c}=0.191$, and NODDI $\rho_{c}=0.569$.

The scatter plots of Figure 4a compare dMRI $D_{\text{a}}$ against its predicted value from histology ${\tilde{D}}_{a}$, Eq. (9), and reveal that $D_{\text{a}}$ from SMI agrees best with the identity line. For the other methods, the estimation of $D_{\text{a}}$ could be biased because of SMT constraint $D^{∥}=D_{\text{a}}=D_{\text{e}}^{∥}$, and WMTI assumption $D_{\text{a}}\leq D_{\text{e}}^{∥}$. NODDI keeps $D_{\text{a}}$ fixed and therefore the scatter plots for this metric are not shown. Correlation between $D_{\text{a}}$ and axon diameter was observed for SMI, but not for the other methods; however, it had the lowest value among the significant correlations of SMI.

Figure 4b reveals positive correlations for SMI and WMTI $D_{\text{e}}^{⊥}$ with histological f, while negative correlations are observed for SMT and NODDI.The negative correlations follow from the extra-axonal tortuosity approximation used by NODDI and SMT (Kaden et al., 2016; Szafer et al., 1995; Zhang et al., 2012), that relates $D_{\text{e}}^{⊥}$ with f and $D_{\text{e}}^{∥}$ as shown in Table 1, and as also observed in Monte Carlo simulation studies that mimic axonal loss (Fieremans et al., 2012; Szafer et al., 1995). SMI and WMTI, on the other hand, do not impose this constraint, and showed a somewhat surprising opposite sign of correlation.

Using the segmented 3d axons, we validated in Figure 5a that the dispersion angle $\theta_{p_{2}}$, computed from the histological FOD $p_{2}$ in Eq. (6), is a good approximation for the true angle of dispersion θ of the axons. Besides dMRI $p_{2}$, and $\theta_{p_{2}}$, SMI also estimates FOD in SH basis using voxel-wise response functions. The rotational invariants $p_{l}$ computed from these dMRI FOD, Eq. (3), correlate with their corresponding histological $p_{l}$, as shown in Figure 5b. These higher-order $p_{l}$ allow us to further validate their exponential decay (Lee et al., 2019; Reisert et al., 2017) with respect to the increasing order l, for l≥2 in Eq. (7), for both 3d EM and dMRI, as shown in Figure 5c for the ipsilateral cingulum of all animals. Furthermore, by comparing the exponential decay parameters C and λ, from SMI and histology, Figure 5d shows better agreement for C than for λ.

dMRI parameters C and λ were estimated from the FOD rotational invariants $p_{2}$, $p_{4}$ and $p_{6}$ obtained from the $p_{lm}$ estimated with SMI, without imposing the exponential decay in Eq. (7) as constraint. In addition, we derived in the Appendix the functional form for $P(\hat{n})$, Eq. (A5), corresponding to Eq. (7), together with the constraint on C and λ imposed by the FOD non-negativity.

**Fig. 6.**
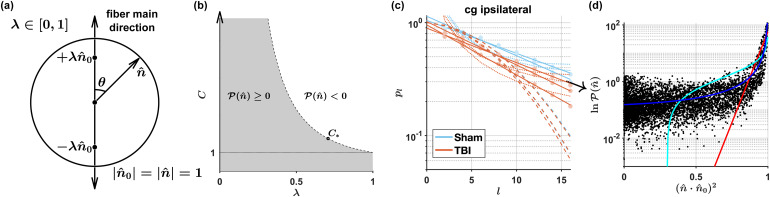
FOD (A5) compatible with Eq. (7). (a) Axially symmetric $P(\hat{n})$, Eq. (A5), whose rotational invariants scale according to Eq. (7), corresponds to the $∼1/r^{3}$ potential induced on a unit sphere $|\hat{n}|\;=1$ by the two identical sources inside the sphere at $\pm \lambda {\hat{n}}_{0}$ along the fiber main direction ${\hat{n}}_{0}$. (b) The domain (A9) of acceptable values (gray) for C and λ, for which $P(\hat{n})\geq 0$ for any unit $\hat{n}$. (c) The exponentially decaying $p_{l}$, Eq. (7), (solid lines) describes the histological FOD invariants $p_{l}$ (points) better than the rotational invariants of the Watson distribution (A14) (dashed) and of the Poisson-kernel FOD (A10) (dotted). (d) FOD (A5) (blue) describes the histological FOD (black points) better than the Watson FOD (red) and the Poisson-kernel FOD (cyan). An example for the FOD corresponding to the $p_{l}$ in (c) is indicated by the arrow.

Using the FOD from the 3d EM samples, in Figure 6 we tested our FOD (A5), as well as previously proposed axially-symmetric FODs—the Watson distribution (Zhang et al., 2012) and the Poisson kernel (Reisert et al., 2017). We found that the FOD (A5) corresponding to Eq. (7) describes the histological FOD well, whereas the functional forms and the rotational invariants of the previously proposed FODs are incompatible with histology away from the main FOD peak.

## 4 Discussion

Biophysical modeling bridges the resolution gap between the macroscopic dMRI voxel and microscopic tissue features, and provides a powerful tool to study development, aging, and diseases at the cellular level where these processes originate. Numerous human *in vivo* studies in brain WM (Beck et al., 2021; Benitez et al., 2014, 2018; Cox et al., 2016; de Kouchkovsky et al., 2016; Fieremans et al., 2013; Fu et al., 2019; Granberg et al., 2017; Hui et al., 2012; Jelescu et al., 2015a; Johnson et al., 2021; Liao et al., 2024; Lynch et al., 2020; Mah et al., 2017; Muller et al., 2021; Palacios et al., 2020; Takeshige-Amano et al., 2022; Wang et al., 2019) have been conducted using SM parameters derived from different estimators: WMTI, NODDI, SMT, and SMI. However, the interpretation of these and future studies relies on the biological accuracy and promised microstructural specificity of the metrics provided by these estimators (Jelescu and Budde, 2017; Novikov, Kiselev, et al., 2018), which highly depends on how correctly they describe the relevant features of the cellular environment, and how accurately and precisely they are estimated.

Previous histological validation efforts for SM metrics have been mainly focused on evaluating their sensitivity, but not their specificity. Indeed, the intra-axonal water fraction f has been evaluated in rodents by comparing against axon density or axon volume fraction metrics derived from 2 d optical and electron microscopy for normal tissue (Jespersen et al., 2010), and models for traumatic brain injury (TBI) (Wang et al., 2013), demyelination (Jelescu et al., 2016), and axonal degeneration (Coronado-Leija et al., 2021); f also has been evaluated in postmortem human tissue for multiple sclerosis (Grussu et al., 2017). However, the use of 2d microscopic images limited the scope of these studies, since 3d tissue properties, such as the FOD and axon morphology metrics (that reveal injuries along the axons), could not be quantified, and the specificity of f with respect to these metrics was not evaluated. Other studies have compared the FOD and/or dispersion parameters against related histological metrics derived, not from segmented axons, but from structure tensor analysis of 2d optical microscopy (Grussu et al., 2017; Leergaard et al., 2010; Mollink et al., 2017), 3d confocal (Schilling et al., 2018) and 3d electron microscopy (Salo et al., 2021). However, the previous 3d studies focused on healthy tissue and did not evaluate any other metric besides dispersion, preventing the validation of the specificity of SM metrics. Furthermore, previous histological works did not assess the differences between the available SM estimators, such as WMTI, NODDI, SMT, and SMI, and focused the analysis on only one of them, with its specific biases in parameter estimation due to given model assumptions. Since all SM estimators are biased in one way or another, especially when using sub-optimal data such as clinically feasible dMRI acquisitions, the biases of each method may become more evident in a study comparing several SM estimators, which might help to gain perspective about their performance, similarities and differences.

In this work, we characterized WM microstructure of sham-operated and TBI rat brains using 3d EM and *ex vivo* dMRI to validate the sensitivity and specificity of the SM parameters. The number of samples that could be collected was limited by the labor-intensive EM tissue preparation, especially for the large 3d EM samples used in this work (Salo et al., 2018). However, thanks to the features of these samples (comparable to the MRI voxel size) and the automated axon segmentation (Abdollahzadeh et al., 2019; Abdollahzadeh, Belevich, et al., 2021), we were able to include a large number of segmented 3d axons per population (in the order of ten thousand) in a diverse set of ROIs (from corpus callosum and cingulum, in the ipsi- and contralateral hemispheres of the brain, for sham-operated and TBI rats), enabling an unprecedented microstructure characterization in comparison with previous histological studies. This histological analysis (Fig. 1e; Supplementary Fig. S2) reveals the following WM microstructure changes due to TBI: reduction in axon density, injuries along the axon (reduction in axon diameter in addition to increase in beading and undulation), and increase in axon dispersion. The simultaneous changes in several microstructural properties indicate the complexity of the damage in chronic TBI, and the need for sensitive and specific dMRI biomarkers in order to properly disentangle them.

The direct comparison of 3d EM derived f versus dMRI intra-axonal water fraction f (Fig. 3) reveals the strongest sensitivity for f estimated with SMT and SMI, followed by WMTI, as well as strong specificity for these estimators, as there are no correlations between f and θ nor $D_{\text{a}}$. On the other hand, f from NODDI correlates with θ but not with histological f, which may suggest that imposing constraints biases NODDI parameter estimation, especially in pathological scenarios (Jelescu et al., 2020). Nonetheless, the otherwise overall agreement between SM estimators in terms of sensitivity and specificity is in line with the consistent findings in many human *in vivo* studies that report changes in f with development, aging, and disease. Indeed, f has been shown to increase in development and decrease in aging using NODDI (Beck et al., 2021; Cox et al., 2016; Jelescu et al., 2015a; Liao et al., 2024; Lynch et al., 2020; Mah et al., 2017), WMTI (Beck et al., 2021; Benitez et al., 2018; Jelescu et al., 2015a; Liao et al., 2024), SMT (Beck et al., 2021; Liao et al., 2024), and SMI (Liao et al., 2024). In neurological disorders, f is observed to increase in stroke ischemic lesions using NODDI (Wang et al., 2019), WMTI (Hui et al., 2012), and SMI (Liao et al., 2024), while decreasing in TBI (Muller et al., 2021; Palacios et al., 2020) using NODDI, Alzheimer’s disease using WMTI (Benitez et al., 2014; Fieremans et al., 2013) and NODDI (Fu et al., 2019), and in Parkinson’s disease (Takeshige-Amano et al., 2022) using NODDI. Furthermore, f has been suggested as a surrogate marker to track disease as it correlates with disability scores in multiple sclerosis, using WMTI (de Kouchkovsky et al., 2016), NODDI, SMT (Johnson et al., 2021), and SMI (Liao et al., 2024). The consistent correspondence between f estimated by WMTI, SMT, and SMI with 3d EM f shows that changes in this metric can be interpreted as mainly driven by changes in axon volume fractions.

For dispersion, Figure 3 shows that the dMRI dispersion angle $\theta_{p_{2}}$ is sensitive and specific to the 3d EM true angle of dispersion of the axons θ for SMI, SMT, and NODDI, while for WMTI it shows sensitivity but not specificity, correlating also with histology f. Our histological validation may help interpret human *in vivo* studies that showed changes in dMRI-derived axon dispersion, namely reduced dispersion in Alzheimer’s disease (Fu et al., 2019), and chronic TBI (Muller et al., 2021), while increased dispersion in stroke (Liao et al., 2024; Wang et al., 2019), multiple sclerosis (Granberg et al., 2017; Liao et al., 2024), and Parkinson’s disease (Takeshige-Amano et al., 2022). The quantitative differences obtained by distinct SM estimators, as shown in Figures 2 and 4a, are also observed in human *in vivo* studies using different estimators. In particular, dispersion has been shown to decrease during neurodevelopment using WMTI (Jelescu et al., 2015a) and SMI (Liao et al., 2024) in the genu corpus callosum, which has been associated with axonal pruning, while weak or no significant changes have been observed using NODDI (Jelescu et al., 2015a; Liao et al., 2024; Lynch et al., 2020; Mah et al., 2017). Our histological validation helps understand these discrepancies and highlights the importance of SM estimators and their underlying constraints. Interestingly, SMI provides a good one-to-one correspondence with histological dispersion angle $\theta_{p_{2}}$, which confirms that the lack of hard constraints (Table 1) in SM estimation results in the best overall accuracy for SMI (Liao et al., 2024).

The large number of segmented axons enabled further validation for the exponential decay, in Eq. (7), of the FOD $p_{l}$, as shown in Figure 5c. In the Appendix, we derived the axially symmetric FOD, in Eq. (A5), corresponding to Eq. (7), and compared it with previously proposed functional forms for the FOD: the Watson distribution (Zhang et al., 2012), and the Poisson kernel (Reisert et al., 2017). As illustrated in Figure 6, we found that the FOD (A5) corresponding to Eq. (7) describes the histological FOD well, whereas the functional forms and the rotational invariants of the Watson distribution and the Poisson kernel do not agree with histology.

Using microscopy data to validate compartmental specific diffusivities $D_{\text{a}}$, $D_{\text{e}}^{∥}$, and $D_{\text{e}}^{⊥}$ is challenging, as there is no straightforward way to directly match them with histological metrics (Jelescu & Budde, 2017). So far, alternative methods for the validation of diffusivities have been proposed such as the use of physical phantoms (Fieremans & Lee, 2018), electrophysiology (Geraldo et al., 2018), metabolites detected through proton magnetic resonance spectroscopy (Kroenke et al., 2004), and the injection of MRI-detectable tracers (Duong et al., 1998). Furthermore, insight can be gained from studying changes in these diffusivities under certain pathological conditions (Budde & Frank, 2010; Hui et al., 2012; Jelescu et al., 2016; Lee et al., 2020), or by performing Monte Carlo simulations (Abdollahzadeh et al., 2023; Budde & Frank, 2010; Lee et al., 2020) in realistic substrates mimicking brain tissue in different (pathological) states. Here, we compared SM $D_{\text{a}}$ against the expected diffusivity from histology ${\tilde{D}}_{a}$ (Fig. 4a and Eq. (9) based on the axon tortuosity $\Lambda_{∥}$ (Abdollahzadeh et al., 2023) and the dispersion from axon undulation $\theta_{\text{u}}$ (Lee et al., 2020). Correlations between $D_{\text{a}}$ and ${\tilde{D}}_{a}$ are observed for SMI, SMT and WMTI. However, specificity is observed only for $D_{a}$ from SMI, as $D_{a}$ from SMT and WMTI both correlate with other non-corresponding histological features. Our result underscore the previously observed dependency of $D_{\text{a}}$ on axon diameter variation and axon beading, obtained using Monte Carlo simulations (Abdollahzadeh et al., 2023; Budde & Frank, 2010; Lee et al., 2020) and human *in vivo* studies (Hui et al., 2012; Lee et al., 2020; Liao et al., 2024).

For the extra-axonal perpendicular diffusivity $D_{\text{e}}^{⊥}$, Figure 4b shows different dependencies on EM-derived volume fraction between the different SM estimators. The positive correlation between histological f and SMI/WMTI $D_{\text{e}}^{⊥}$, contradicts the extra-axonal tortuosity approximation used as a hard constraint in NODDI and SMT. While intuitively, $D_{\text{e}}^{⊥}$ is expected to decrease with increasing f, Monte Carlo simulations of axon loss have also shown opposite trends (Fieremans et al., 2012; Szafer et al., 1995). The behavior of $D_{\text{e}}^{∥}$ and $D_{\text{e}}^{⊥}$ during pathological conditions is still an ongoing topic of investigation, as the complex processes happening in the extra-axonal space are not fully understood. Our results suggest that using functional relations between $D_{\text{e}}^{⊥}$ and f is unjustified, and both parameters should rather be estimated independently, particularly in pathological conditions. Interestingly, for SMI and WMTI, that do not employ the tortuosity constraint, several human *in vivo* studies have found changes in their $D_{\text{e}}^{⊥}$ for neurodevelopment (Jelescu et al., 2015a; Liao et al., 2024), aging (Beck et al., 2021; Benitez et al., 2018), stroke (Hui et al., 2012; Liao et al., 2024), Alzheimer’s disease (Benitez et al., 2014; Fieremans et al., 2013), and multiple sclerosis (de Kouchkovsky et al., 2016; Liao et al., 2024).

The extrapolation of results (Schilling et al., 2022) from *ex vivo* dMRI animal data for interpretation of *in vivo* dMRI human data should be done with care, as there are intrinsic differences in tissue properties and MRI acquisition protocol. However, the SM assumptions by design also apply for *in vivo* dMRI data, and a recent study (Liao et al., 2024) has evaluated the wide applicability of SM estimators *in vivo* using common two-shell dMRI protocols (Casey et al., 2018; Glasser et al., 2016; Jack et al., 2008; Miller et al., 2016) for the study of development, aging and pathology (multiple sclerosis and stroke) and demonstrated that the SM parameters reflect the cellular changes anticipated in these conditions.

The main differences between the MRI data used in our study and large human brain data sets (Casey et al., 2018; Glasser et al., 2016; Jack et al., 2008; Miller et al., 2016) are: resolution, diffusion time, and b-values. The high resolution of our data was necessary as rat brains are comparably smaller than human brains. The diffusion time in our work was considerably smaller than typical times used for *in vivo* acquisitions, which may bias the estimation of $D_{\text{a}}$, $D_{\text{e}}^{∥}$ and $D_{\text{e}}^{⊥}$. However, this bias would decrease at longer diffusion times, resulting in improved performance of SM for *in vivo* studies. Lastly, the b-values employed in this work, 2, 3, and 4 ms/μm^2^, were adjusted to the intrinsically lower diffusivities for *ex vivo* MRI acquired at room temperature, and therefore higher than the b-values used for *in vivo* studies at body temperature (Schilling et al., 2022), but equivalent to 1, 1.5, and 2 ms/μm^2^ for *in vivo* MRI. We also used three b-values, one more than commonly used *in vivo* dMRI protocols (Casey et al., 2018; Glasser et al., 2016; Jack et al., 2008; Miller et al., 2016) with shells of b=1 and 2 ms/μm^2^, but repeating the analysis removing the shell with b=3 ms/μm^2^ (equivalent to b=1.5 ms/μm^2^ in vivo) demonstrated similar results (Supplementary Fig. S7).

The use of fixed tissue should also be considered when extrapolating our results to *in vivo* studies. Tissue fixation may modify the morphology of cellular structures by shrinking the extra-cellular space (Cragg, 1980; Korogod et al., 2015) and thereby impact the estimation of the extra-axonal parameters, such as $D_{\text{e}}^{∥}$ and $D_{\text{e}}^{⊥}$. However, we did not make specific conclusions about these parameters besides highlighting the need to release constraints on their estimation. The introduction of more advanced fixation and sample preparation methods might aid in preserving tissue microstructure closer to *in vivo* conditions in future studies (Korogod et al., 2015; Lu et al., 2023).

While our MRI ROIs were larger than the histological volumes (Supplementary Fig. S1), the SM parameters along the tracts (corpus callosum and cingulum) within an ROI showed lower variability (Fig. 2) as compared to the relatively large change due to TBI condition. As we do not know and therefore could not control for the relative fraction between the cingulum and corpus callosum inside crossing fiber voxels, we evaluated this potential confounding effect by performing the analyses including only voxels in the ROIs with crossings that match the EM well (Supplementary Fig. S9), and found similar results as compared to including all voxels in the ROI (in Fig. 3). These additional analyses (Supplementary Figs. S7 and S8) support the robustness of our *ex vivo* histological validation study.

In conclusion, this work bridges the gap between micrometer-scale cellular architecture and the macroscopic MRI resolution, by comprehensively validating a key white matter diffusion MRI model and its four publicly available estimators, WMTI, NODDI, SMT, and SMI. The uniqueness of the present approach is in the 3d EM, the use of pathology (TBI) as well as sham-operated animals, the variety of the samples, and the large number of segmented 3d axons. We demonstrate the correspondence between histology and SM parameters, for which SMI showed the highest sensitivity and specificity.

The validated Standard Model may become a powerful tool for neuroscience research, and provide valuable non-invasive imaging markers to study brain development, aging, disease, disorders, and injuries at the cellular level where these processes originate.

## Data and Code Availability

The 3d EM volumes and their segmentation are publicly available at https://etsin.fairdata.fi/dataset/f8ccc23a-1f1a-4c98-86b7-b63652a809c3. The dMRI volumes and the ROIs are publicly available at https://etsin.fairdata.fi/dataset/29e410dc-5d58-45ac-a716-ce1a38b46037. Several publicly available toolboxes were used in this study as stated in the Section 2. Extra in-house MATLAB scripts created for the analysis of the 3d EM and dMRI are publicly available at https://github.com/NYU-DiffusionMRI/Standard-Model-validation-using-3d-EM.

## Author Contributions

R.C.L., E.F., and D.S.N. conceived the project and designed the study. R.C.L. performed the formal analysis of the 3d EM and dMRI data, extracted the microstructural metrics, and compared them. A.A., J.T., and A.S. processed and segmented the 3d EM data. A.S. provided animal models, dMRI and EM imaging. H.H.L., S.C., B.A.A., and Y.L. provided software for the processing and analysis of the EM and dMRI data. R.A.S. assisted in the analysis of the dMRI data. R.C.L. and D.S.N. derived and analyzed the FOD functional form. R.C.L. and E.F. wrote the manuscript. E.F. and D.S.N. supervised the project. All authors commented, edited, and approved the final manuscript.

## Funding

Research was supported by the National Institute of Neurological Disorders and Stroke of the NIH under awards R01 NS088040 and R21 NS081230, and by the Irma T. Hirschl fund, and was performed at the Center of Advanced Imaging Innovation and Research (CAI2R, www.cai2r.net), a Biomedical Technology Resource Center supported by NIBIB with the award P41 EB017183. A.S. was funded by the Academy of Finland (grant #323385) and the Erkko Foundation. H.H.L. was funded by the Office of the Director of the NIH and the National Institute of Dental and Craniofacial Research of the NIH under award DP5 OD031854.

## Declaration of Competing Interest

E.F. and D.S.N. are co-inventors in technology related to this research with US patents US10360472B2 and US10698065B2.

## Supplementary Materials

Supplementary material for this article is available with the online version here: https://doi.org/10.1162/imag_a_00212.

## Supplementary Material

Supplementary Material

## Appendix: Fod Functional Form

Here, we analytically derive the FOD based on its rotational invariants $p_{l}$ following the exponential decay, Eq. (7), under an additional assumption of axial symmetry, and compare with previously proposed axially symmetric FOD functional forms: the Watson distribution (Zhang et al., 2012) and the Poisson kernel (Reisert et al., 2017).

Assuming the fiber oriented along ${\hat{n}}_{0}$, and keeping only the m=0 SH terms in the ${\hat{n}}_{0}$ basis, Eqs. (3)–(5) yield

P(n^)=∑l=0,2,...pl0Yl0(n^⋅n^0)=∑l=0,2,...Nlpl⋅Nl4πPl(n^⋅n^0)=∑l=0,2,...(2l+1)plPl(n^⋅n^0).
(A1)

To derive $P(\hat{n})$ corresponding to Eq. (7), we substitute $p_{l}\to C\lambda^{l}+\left(1-C\right)\delta_{l0}$, where the $\delta_{l0}$ term (Lee et al., 2019) ensures the normalization $p_{0}=1$:

$$P\left(\hat{n}\right)=\left(2\lambda \partial_{\lambda }+1\right)C\sum_{l\;=\;0,\;\;2,\;...}\lambda^{l}P_{l}\left(\hat{n}\cdot {\hat{n}}_{0}\right)+1-C,$$
(A2)

where (Novikov, Veraart, et al., 2018) $\left(2l+1\right)\;\lambda^{l}=\left(2\lambda \partial_{\lambda }+1\right)$$\lambda^{l}$. Given the Poisson kernel (Reisert et al., 2017) (Coulomb potential of a unit charge at $\lambda {\hat{n}}_{0}$)

$$\sum_{l=0}^{\infty }\lambda^{l}P_{l}\left(\hat{n}\cdot {\hat{n}}_{0}\right)=\frac{1}{\sqrt{1-2\lambda \hat{n}\cdot {\hat{n}}_{0}+\lambda^{2}}}=\frac{1}{\left|\hat{n}-\lambda {\hat{n}}_{0}\right|},$$
(A3)

we add the charge at $-\lambda \hat{n}$ to ensure the antipodal symmetry ($l_{\text{odd}}=0$), and obtain the sum in Eq. (A2):

$$\sum_{l\;=\;0,\;2,\;...}\lambda^{l}P_{l}\left(\hat{n}\cdot {\hat{n}}_{0}\right)=\frac{1/2}{\left|\hat{n}-\lambda {\hat{n}}_{0}\right|}+\frac{1/2}{\left|\hat{n}+\lambda {\hat{n}}_{0}\right|}.$$
(A4)

Applying $2\lambda \partial_{\lambda }+1$ to Eq. (A4), we finally obtain

$$P\left(\hat{n}\right)=C\left[\frac{1-\lambda^{2}}{2}\left(\frac{1}{{\left|\hat{n}-\lambda {\hat{n}}_{0}\right|}^{3}}+\frac{1}{{\left|\hat{n}+\lambda {\hat{n}}_{0}\right|}^{3}}\right)-1\right]+1.$$
(A5)

Geometrically, the FOD (A5) corresponds to the $∼1/r^{3}$ potential from two identical sources inside the unit sphere at $\pm \lambda n_{0}$, induced on the sphere’s surface $|\hat{n}|\;=1$, Figure 6a.

The domain of acceptable values of C and λ is found from the condition $P(\hat{n})\geq 0$ for any $\hat{n}$. By symmetry, the minimal value of the expression in braces in Eq. (A5) is attained at the equator, $\hat{n}\cdot {\hat{n}}_{0}=0$, therefore

$$\text{min}\;\;P(\hat{n})=C\frac{1-\lambda^{2}}{{\left(1+\lambda^{2}\right)}^{3/2}}+1-C\geq 0.$$
(A6)

For C≤1, that is, when the FOD is dominated by its constant $p_{0}=1$ term, the condition in Eq. (A6) holds for any 0≤λ≤1. When C>1, the −1 term in the square brackets in Eq. (A5) can make the FOD negative. Indeed, the substitution $z_{\lambda }^{2}=1/\left(1+\lambda^{2}\right)$ turns the above condition into the “depressed cubic” form

$$z_{\lambda }^{3}+pz_{\lambda }\geq q,\;p=-\frac{1}{2},\;q=\frac{C-1}{2C}.$$
(A7)

The FOD condition in Eqs. (A6)–(A7) holds for q≤0, that is, for C≤1, since $z_{\lambda }\left(z_{\lambda }^{2}-1/2\right)\geq 0$ for $1/\;\sqrt{2}\leq z_{\lambda }\leq 1$ corresponding to the relevant range 0≤λ≤1. This condition, however, can break down when q>0, for which C>1. According to the Cardano formula, when $D\left(C\right)=q^{2}/4$$+p^{3}/27>0$, corresponding to $C>C_{*}=1/\left(1-\zeta \right)\approx 1.374$, where $\zeta =\frac{1}{3}\sqrt{\frac{2}{3}}$, the cubic in Eq. (A7) has one real root, and the FOD is non-negative when

$$z_{\lambda }\geq z\left(C\right)={\left(q/2+\sqrt{D}\right)}^{1/3}+{\left(q/2-\sqrt{D}\right)}^{1/3}.$$
(A8)

In the opposite case, $1<C<C_{*}$, D(C)<0, and there are three real roots given by the Vieta formula. It turns out that the FOD non-negativity is determined by the largest one, which coincides with Eq. (A8), where $\pm \sqrt{D}$ are now purely imaginary and complex-conjugate. All together, the condition (A8) translates into

$$0\leq \lambda \leq \;\{\begin{array}{c} 1,\\ \sqrt{\frac{1}{z^{2}\left(C\right)}\;-1,} \end{array}\begin{array}{c} C\leq 1;\\ C>1, \end{array}$$
(A9)

defining the shaded domain in Figure 6b.

The antipodally-symmetric Poisson kernel (A4), characterized by a single parameter λ, has been suggested previously as an FOD functional form (Reisert et al., 2017). To compare with Eq. (A5) and to decouple the l=0 and l>0 sectors, we generalize it to

$$P_{\text{Poiss}}\left(\hat{n}\right)=C\left(\frac{1/2}{\left|\hat{n}-\lambda {\hat{n}}_{0}\right|}+\frac{1/2}{\left|\hat{n}+\lambda {\hat{n}}_{0}\right|}-1\right)+1.$$
(A10)

Comparing Eqs. (A1) and (A3), we find

$$\left(2l+1\right)p_{\text{Poiss},l}=C\lambda^{l}+\left(1-C\right)\delta_{l0}.$$
(A11)

The condition $P_{\text{Poiss}}(\hat{n})\geq 0$ for any $\hat{n}$ can also be found by looking at the equator, $\hat{n}\cdot {\hat{n}}_{0}=0$, where

$$\text{min}\;P_{\text{Poiss}}\left(\hat{n}\right)=\frac{C}{\sqrt{1+\lambda^{2}}}+1-C\geq 0.$$
(A12)

This always holds for 0≤C≤1, while for C>1, the Poisson-kernel FOD is non-negative when

$$0\leq \lambda \leq \frac{\sqrt{2C-1}}{C-1},\;\;C>1.$$
(A13)

Popular Standard Model estimators, such as NODDI (Zhang et al., 2012) and WMTI-Watson (Jespersen et al., 2018), represent the FOD via the Watson distribution

$$P_{\text{Watson}}\left(\hat{n}\right)=M\left(\frac{1}{2},\;\frac{3}{2},\kappa \right)e^{\kappa {\left(\hat{n}\cdot {\hat{n}}_{0}\right)}^{2}},$$
(A14)

where M is a confluent hypergeometric function ensuring the FOD normalization, and κ is the concentration parameter. In the main text, we use the relation (Jespersen et al., 2018)

$$p_{2}\left(\kappa \right)=\frac{1}{4}\left(\frac{3}{\sqrt{\kappa }F\left(\sqrt{\kappa }\right)}-2-\frac{3}{\kappa }\right),$$
(A15)

where $F\left(x\right)=\frac{\pi }{2}e^{-x^{2}}\text{erfi}\left(x\right)$ is the Dawson’s function. Using the orthogonality of Legendre polynomials, we analytically derived

$$p_{l}\left(\kappa \right)=\int_{0}^{1}\text{d}x\;P_{l}\left(x\right)M\left(\frac{1}{2},\frac{3}{2},\kappa \right)e^{\kappa x^{2}}$$
(A16)

up to l=16 (cumbersome expressions not shown).

Figure 6 compares the derived FOD (A5), the Poisson-kernel FOD (A10), and the Watson FOD (A14) to histology. Based on the data from the ipsilateral cingulum for all animals, Figure 6c shows that the histological FOD rotational invariants $p_{l}$ are better described by the exponential decay (7), corresponding to Eq. (A5) (solid straight lines), than by $p_{l}(\kappa )$ from the Watson FOD (dashed), Eq. (A16), yielding the concave curves. For the Poisson-kernel FOD (dotted), the rotational invariants $p_{\text{Poiss},l}∼\lambda^{l}/\left(2l+1\right)$ according to Eq. (A11) are convex in the semi-logarithmic plot, hence they also do not exhibit the scaling compatible with the histological FOD rotational invariants $p_{l}$. Our previous study in Lee et al. (2019) missed the factor 2l+1 and misinterpreted the exponentially decaying $p_{l}$, Eq. (7), as validation for the Poisson-kernel FOD hypothesized in Reisert et al. (2017).

Similarly, Figure 6d shows that Eq. (A5) describes the full shape of the histological FOD better than the Watson distribution (A14), and the Poisson-kernel FOD (A10). Here, κ for the Watson distribution (A14) was estimated non-linearly from the histology FOD points (Fig. 6d), and the corresponding $p_{l}(\kappa )$ were computed analytically via Eq. (A16) and plotted in Figure 6c, while parameters C and λ for the two other FODs were linearly estimated from the corresponding rotational invariants $p_{l}$ with l>0 (Fig. 6c), and substituted into Eqs. (A10) and (A5), respectively (generating the cyan and blue curves in Fig. 6d).

As a result, the present analysis shows that the derived FOD (A5) represents the data well, whereas neither the Watson FOD (A14) nor the Poisson-kernel FOD (A10) for any C is compatible with histology outside of the vicinity of the FOD peak.
