# Supplementary material for: Volume electron microscopy in injured rat brain validates white matter microstructure metrics from diffusion MRI
Source: Imaging Neurosci (Camb). 2024 Jul 2;2:imag-2-00212. doi: 10.1162/imag_a_00212 (PMC12272244; doi:10.1162/imag_a_00212)
Supplement: Supplementary Material [file imag_a_00212-supp.pdf]

Supplementary Information

Volume electron microscopy in injured rat brain validates white matter microstructure metrics from diffusion MRI

Ricardo Coronado-Leija[1,*], Ali Abdollahzadeh[1], Hong-Hsi Lee[2], Santiago Coelho[1], Benjamin Ades-Aron[1], Raimo A. Salo[3], Jussi Tohka[3], Alejandra Sierra[3], Dmitry S. Novikov[1], Els Fieremans[1]

[1]Bernard and Irene Schwartz Center for Biomedical Imaging, Department of Radiology, New York University School of Medicine, New York, NY, USA
[2]Athinoula A. Martinos Center for Biomedical Imaging, Department of Radiology, Massachusetts General Hospital, Harvard Medical School, Boston, MA, USA
[3]A.I. Virtanen Institute for Molecular Sciences, University of Eastern Finland, Kuopio, Finland
[*]ricardo.coronadoleija@nyulangone.org, rleija@cimat.mx

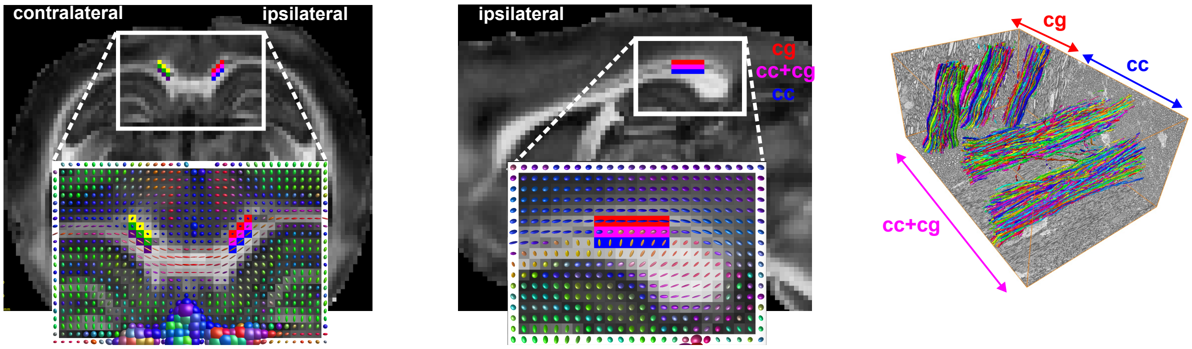

FIG. S1. Six ROIs were analyzed on each animal: cc, cg and cc+cg for the ipsi- and contralateral hemispheres.

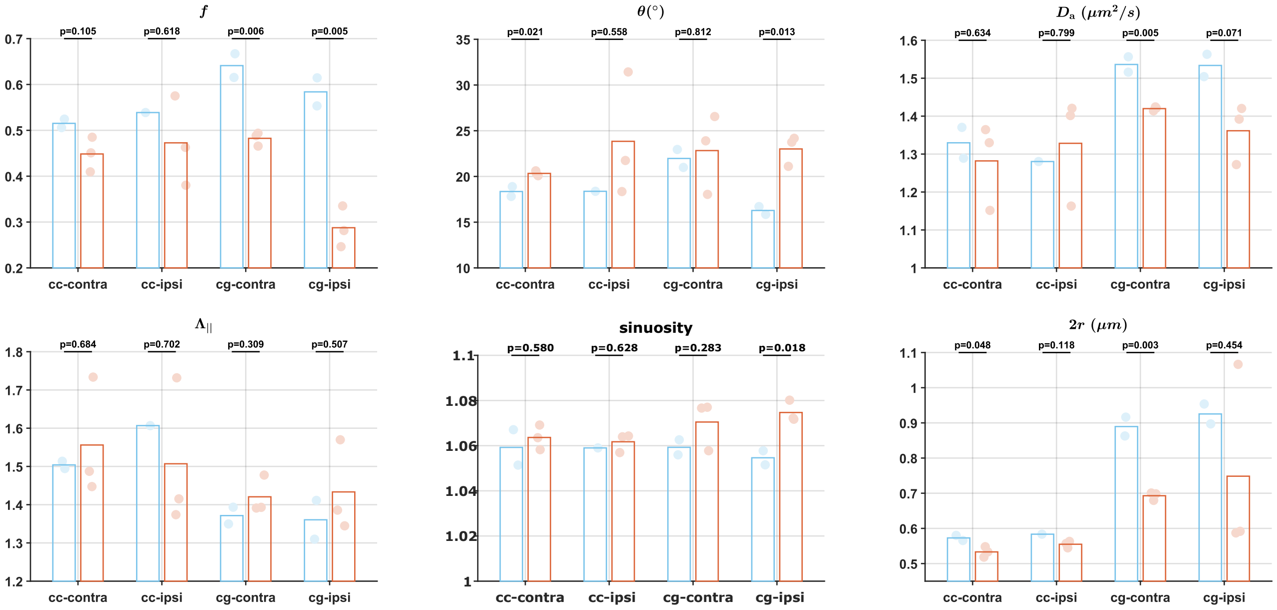

FIG. S2. Comparison between TBI and sham-operated animals, on each tract, for the 3d EM derived metrics. P-values were derived using a t-test.

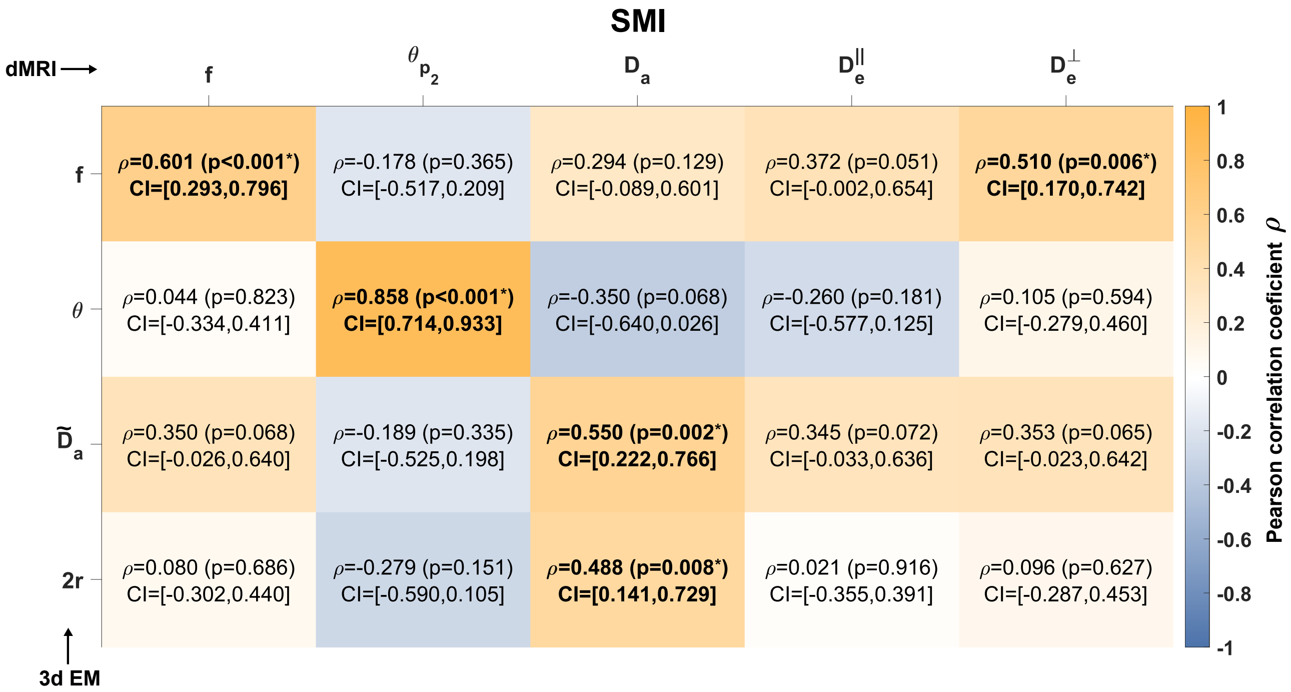

FIG. S3. **Comparison between SMI parameters and 3d EM derived metrics**. Each cell shows the Pearson correlations coefficient, p-values and 95% confidence intervals. Significant correlations ($p < 0.05$) are highlighted in bold, where * indicates that significance remains after adjusting for multiple comparisons using the false discovery rate.

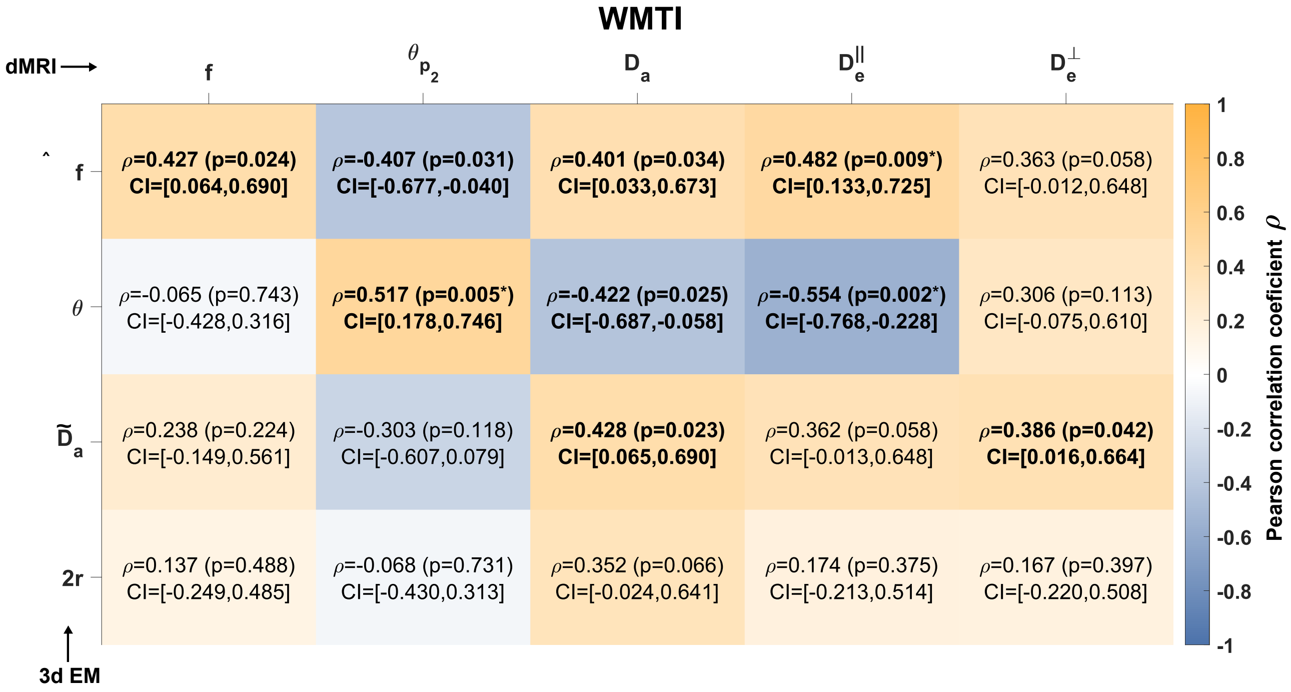

FIG. S4. **Comparison between WMTI parameters and 3d EM derived metrics**. Each cell shows the Pearson correlations coefficient, p-values and 95% confidence intervals. Significant correlations ($p < 0.05$) are highlighted in bold, where * indicates that significance remains after adjusting for multiple comparisons using the false discovery rate.

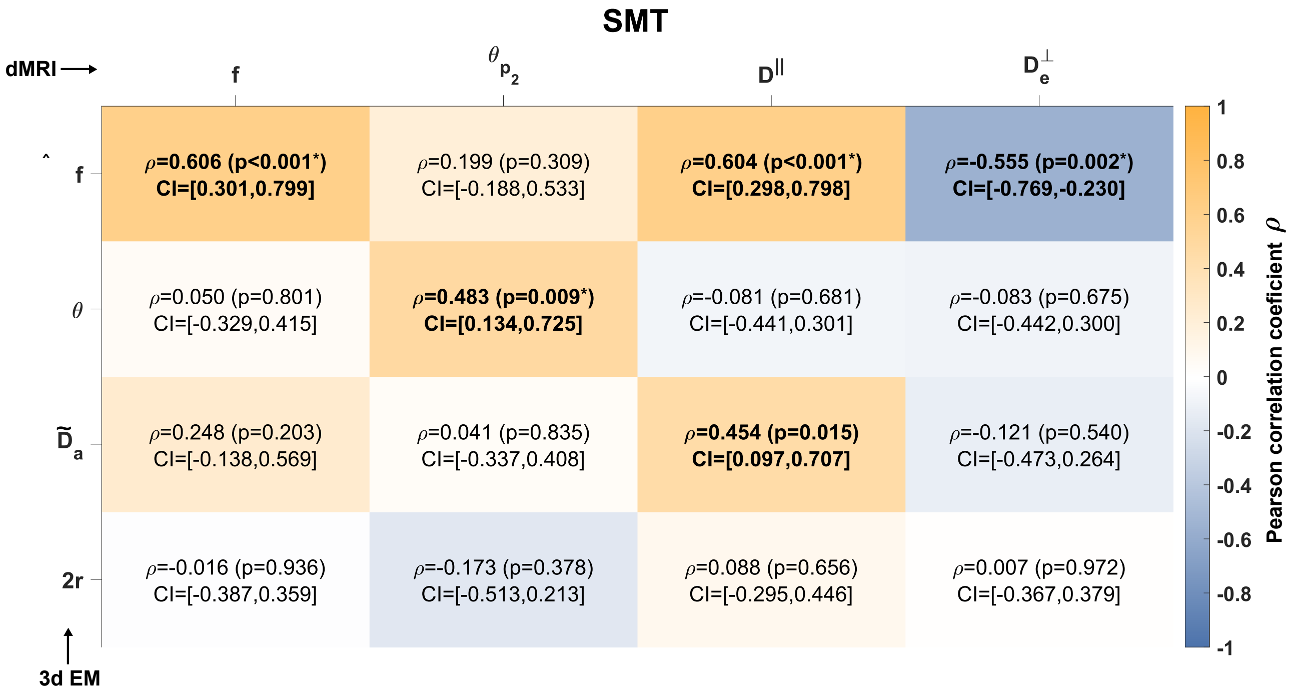

FIG. S5. **Comparison between SMT parameters and 3d EM derived metrics**. Each cell shows the Pearson correlations coefficient, p-values and 95% confidence intervals. Significant correlations ($p < 0.05$) are highlighted in bold, where * indicates that significance remains after adjusting for multiple comparisons using the false discovery rate.

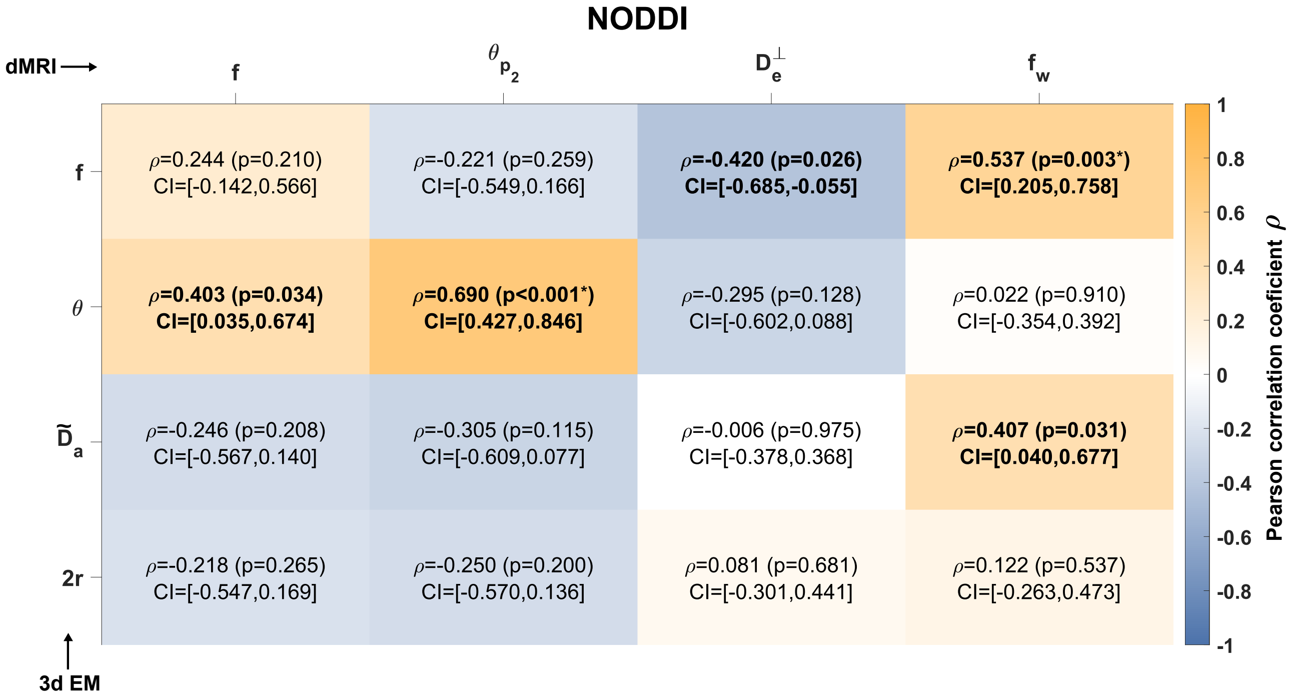

FIG. S6. **Comparison between NODDI parameters and 3d EM derived metrics**. Each cell shows the Pearson correlations coefficient, p-values and 95% confidence intervals. Significant correlations ($p < 0.05$) are highlighted in bold, where * indicates that significance remains after adjusting for multiple comparisons using the false discovery rate.

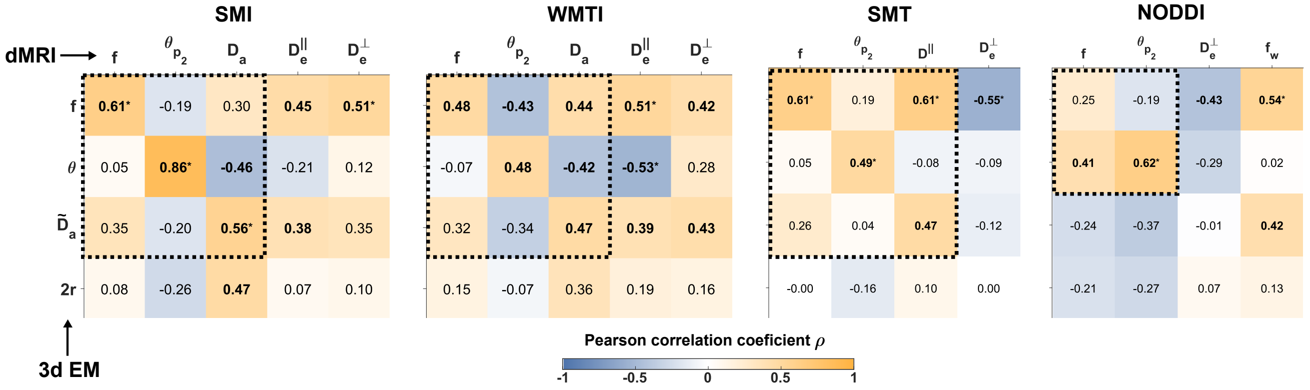

FIG. S7. **Comparison between two-shell dMRI and 3d EM derived metrics**. By removing the shell with $b = 3$ ms/µm^2 from the dMRI data before computing the SM parameters, we observe good reproducibility of the results in Fig. 3, particularly for NODDI and SMT, while a few extra significant non-corresponding correlations are observed for SMI and WMTI, which do not survive multiple-comparison correction.

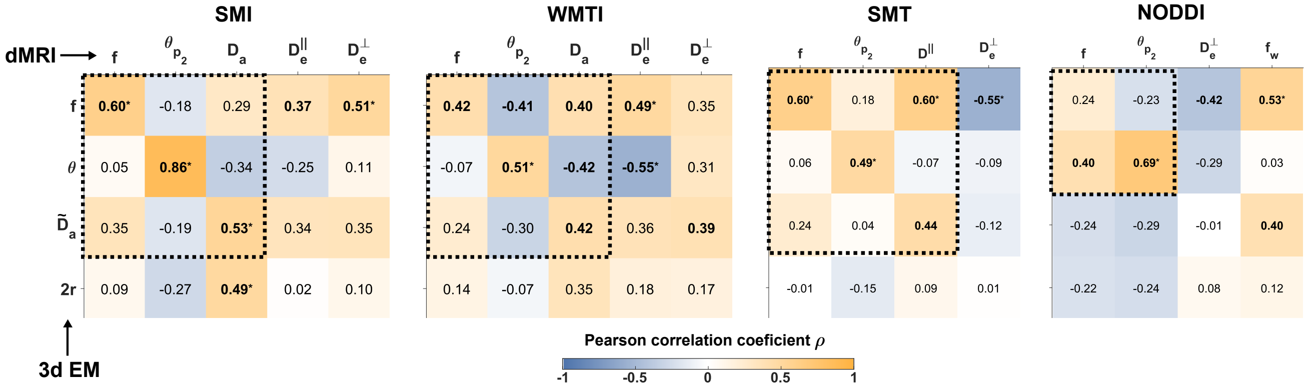

FIG. S8. **Comparison between dMRI and 3d EM derived metrics using bootstrapping and permutation testing**. In order to account for non-Gaussianity and the small sample size, we re-computed the Pearson correlation coefficients and the p-values, in Fig. 3, using bootstrapping and permutation testing, respectively. We observe similar results than Fig. 3.

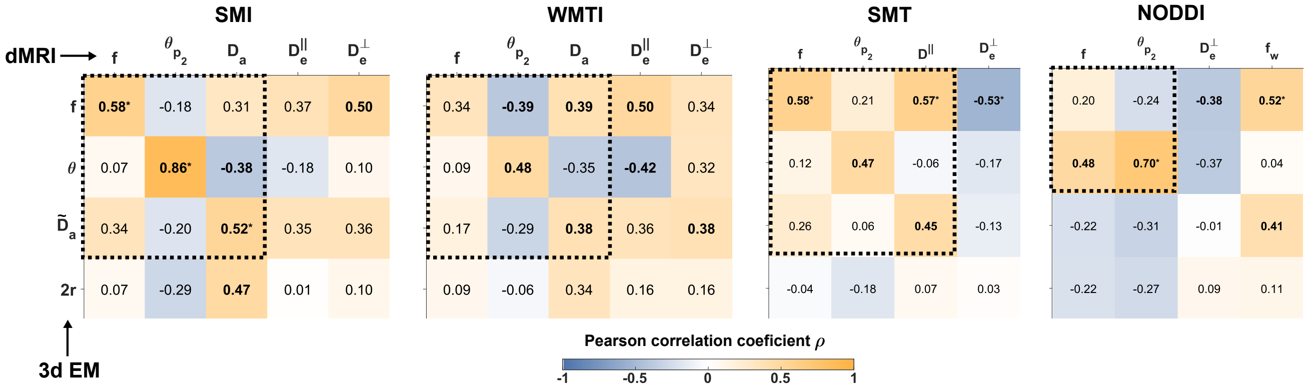

FIG. S9. **Comparison between dMRI and 3d EM derived metrics with selected crossing fiber voxels**. To better match histological microstructure on crossing fiber ROIs, we repeated the analysis using only voxels in which the FOD lobe corresponding to the corpus callosum was larger than the cingulum. We observe similar results than Fig. 3, particularly for SMI, SMT and NODDI.
